# Genetic analysis in European ancestry individuals identifies 517 loci associated with liver enzymes

**DOI:** 10.1038/s41467-021-22338-2

**Published:** 2021-05-10

**Authors:** Raha Pazoki, Marijana Vujkovic, Joshua Elliott, Evangelos Evangelou, Dipender Gill, Mohsen Ghanbari, Peter J. van der Most, Rui Climaco Pinto, Matthias Wielscher, Matthias Farlik, Verena Zuber, Robert J. de Knegt, Harold Snieder, André G. Uitterlinden, Lifelines Cohort Study, H. Marike Boezen, Lude Franke, Pim van der Harst, Gerjan Navis, Marianne Rots, Morris Swertz, Bruce H. R. Wolffenbuttel, Cisca Wijmenga, Julie A. Lynch, Xiyun Jiang, Saredo Said, David E. Kaplan, Kyung Min Lee, Marina Serper, Rotonya M. Carr, Philip S. Tsao, Stephen R. Atkinson, Abbas Dehghan, Ioanna Tzoulaki, M. Arfan Ikram, Karl-Heinz Herzig, Marjo-Riitta Järvelin, Behrooz Z. Alizadeh, Christopher J. O’Donnell, Danish Saleheen, Benjamin F. Voight, Kyong-Mi Chang, Mark R. Thursz, Paul Elliott, the VA Million Veteran Program, Zuhair K. Ballas, Sujata Bhushan, Edward J. Boyko, David M. Cohen, John Concato, Michaela Aslan, Hongyu Zhao, Joseph I. Constans, Louis J. Dellitalia, Joseph M. Fayad, Ronald S. Fernando, Hermes J. Florez, Melinda A. Gaddy, Saib S. Gappy, Gretchen Gibson, Michael Godschalk, Jennifer A. Greco, Samir Gupta, Salvador Gutierrez, Kimberly D. Hammer, Mark B. Hamner, John B. Harley, Adriana M. Hung, Mostaqul Huq, Robin A. Hurley, Pran R. Iruvanti, Douglas J. Ivins, Frank J. Jacono, Darshana N. Jhala, Laurence S. Kaminsky, Jon B. Klein, Suthat Liangpunsakul, Jack H. Lichy, Jennifer Moser, Grant D. Huang, Sumitra Muralidhar, Stephen M. Mastorides, Roy O. Mathew, Kristin M. Mattocks, Rachel McArdle, Paul N. Meyer, Laurence J. Meyer, Jonathan P. Moorman, Timothy R. Morgan, Maureen Murdoch, Olaoluwa O. Okusaga, Kris-Ann K. Oursler, Nora R. Ratcliffe, Michael I. Rauchman, R. Brooks Robey, George W. Ross, Richard J. Servatius, Satish C. Sharma, Scott E. Sherman, Elif Sonel, Peruvemba Sriram, Todd Stapley, Robert T. Striker, Neeraj Tandon, Gerardo Villareal, Agnes S. Wallbom, John M. Wells, Jeffrey C. Whittle, Mary A. Whooley, Peter W. Wilson, Yan V. Sun, Junzhe Xu, Shing-Shing Yeh, Todd Connor, Dean P. Argyres, Elizabeth R. Hauser, Jean C. Beckham, Brady Stephens, Samuel M. Aguayo, Sunil K. Ahuja, Saiju Pyarajan, Kelly Cho, J. Michael Gaziano, Scott Kinlay, Xuan-Mai T. Nguyen, Jessica V. Brewer, Mary T. Brophy, Nhan V. Do, Donald E. Humphries, Luis E. Selva, Shahpoor Shayan, Stacey B. Whitbourne, Jim L. Breeling, J. P. Casas Romero, Rachel B. Ramoni

**Affiliations:** 1 Department of Epidemiology and Biostatistics, MRC Centre for Environment and Health, School of Public Health, London, UK; 2 Division of Biomedical Sciences, Department of Life Sciences, College of Health, Medicine and Life Sciences, Brunel University London, Uxbridge, UK; 3 Corporal Michael J. Crescenz VA Medical Center, Philadelphia, PA USA; 4 Perelman School of Medicine, University of Pennsylvania, Philadelphia, PA USA; 5 Royal Surrey County Hospital, Guildford, Surrey UK; 6 Department of Hygiene and Epidemiology, University of Ioannina Medical School, Ioannina, Greece; 7 British Heart Foundation Centre of Research Excellence, Imperial College London, London, UK; 8 Department of Epidemiology, Erasmus University Medical Center Rotterdam, Rotterdam, The Netherlands; 9 Department of Genetics, School of Medicine, Mashhad University of Medical Sciences, Mashhad, Iran; 10 Department of Epidemiology, University of Groningen, University Medical Center Groningen, Groningen, The Netherlands; 11 UK Dementia Research Institute, Imperial College London, London, UK; 12 Department of Dermatology, Medical University of Vienna, Vienna, Austria; 13 Department of Gastroenterology and Hepatology, Erasmus University Medical Center Rotterdam, Rotterdam, The Netherlands; 14 Department of Internal Medicine, Erasmus University Medical Center Rotterdam, Rotterdam, The Netherlands; 15 VA Salt Lake City Health Care System, Salt Lake City, UT USA; 16 University of Massachusetts, Boston, MA USA; 17 School of Medicine, University of Utah, Salt Lake City, UT USA; 18 VA Palo Alto Health Care System, Palo Alto, CA USA; 19 School of Medicine, Stanford University, Stanford, CA USA; 20 Division of Digestive Diseases, Department of Metabolism, Digestion & Reproduction, Imperial College London, London, UK; 21 Institute of Biomedicine, Medical Research Center Oulu, Oulu University, Oulu, Finland; 22 Oulu University Hospital, Oulu, Finland; 23 Institute of Pediatrics, Poznan University of Medical Sciences, Poznan, Poland; 24 Center for Life Course Health Research, Faculty of Medicine, Oulu University, Oulu, Finland; 25 Department of Life Sciences, College of Health and Life Sciences, Brunel University London, Uxbridge, UK; 26 Unit of Primary Care, Oulu University Hospital, Oulu, Finland; 27 VA Boston Healthcare System, Boston, MA USA; 28 Harvard Medical School, Boston, MA USA; 29 Brigham Women’s Hospital, Boston, MA USA; 30 Departments of Medicine and Cardiology, Columbia University, New York City, NY USA; 31 Department of Systems Pharmacology and Translational Therapeutics, Perelman School of Medicine, University of Pennsylvania, Philadelphia, PA USA; 32 Department of Genetics, Perelman School of Medicine, University of Pennsylvania, Philadelphia, PA USA; 33 Institute for Translational Medicine and Therapeutics, Perelman School of Medicine, University of Pennsylvania, Philadelphia, PA USA; 34 National Institute for Health Research, Imperial Biomedical Research Centre, Imperial College London, London, UK; 35 Health Data Research UK at Imperial College London, London, UK; 36 Department of Genetics, University of Groningen, University Medical Center Groningen, Groningen, The Netherlands; 37 Department of Cardiology, University of Groningen, University Medical Center Groningen, Groningen, The Netherlands; 38 Division of Nephrology, Department of Internal Medicine, University of Groningen, University Medical Center Groningen, Groningen, The Netherlands; 39 Department of Pathology and Medical Biology, University of Groningen, University Medical Center Groningen, Groningen, The Netherlands; 40 Department of Endocrinology, University of Groningen, University Medical Center Groningen, Groningen, The Netherlands; 41 Iowa City VA Health Care System, Iowa City, IA USA; 42 VA North Texas Health Care System, Dallas, TX USA; 43 VA Puget Sound Health Care System, Seattle, WA USA; 44 Portland VA Medical Center, Portland, OR USA; 45 VA Connecticut Healthcare System, West Haven, CT USA; 46 Southeast Louisiana Veterans Health Care System, New Orleans, LA USA; 47 Birmingham VA Medical Center, Birmingham, AL USA; 48 VA Southern Nevada Healthcare System, North Las Vegas, NV USA; 49 VA Loma Linda Healthcare System, Loma Linda, CA USA; 50 Miami VA Health Care System, Miami, FL USA; 51 VA Eastern Kansas Health Care System, Leavenworth, KS USA; 52 John D. Dingell VA Medical Center, Detroit, MI USA; 53 Fayetteville VA Medical Center, Fayetteville, AR USA; 54 Richmond VA Medical Center, Richmond, VA USA; 55 Sioux Falls VA Health Care System, Sioux Falls, SD USA; 56 VA San Diego Healthcare System, San Diego, CA USA; 57 Edward Hines Jr. VA Medical Center, Hines, IL USA; 58 Fargo VA Health Care System, Fargo, ND USA; 59 Ralph H. Johnson VA Medical Center, Charleston, SC USA; 60 Cincinnati VA Medical Center, Cincinnati, OH USA; 61 VA Tennessee Valley Healthcare System, Nashville, TN USA; 62 VA Sierra Nevada Health Care System, Reno, NV USA; 63 W.G. (Bill) Hefner VA Medical Center, Salisbury, NC USA; 64 Hampton VA Medical Center, Hampton, VA USA; 65 Eastern Oklahoma VA Health Care System, Muskogee, OK USA; 66 VA Northeast Ohio Healthcare System, Cleveland, OH USA; 67 Philadelphia VA Medical Center, Philadelphia, PA USA; 68 VA Health Care Upstate New York, Albany, NY USA; 69 Louisville VA Medical Center, Louisville, KY USA; 70 Richard Roudebush VA Medical Center, Indianapolis, IN USA; 71 Washington DC VA Medical Center, Washington, DC USA; 72 James A. Haley Veterans Hospital, Tampa, FL USA; 73 Columbia VA Health Care System, Columbia, SC USA; 74 Central Western Massachusetts Healthcare System, Leeds, MA USA; 75 Bay Pines VA Healthcare System, Bay Pines, FL USA; 76 Southern Arizona VA Health Care System, Tucson, AZ USA; 77 VA Salt Lake City Health Care System, Salt Lake City, UT USA; 78 James H. Quillen VA Medical Center, Johnson City, TN USA; 79 VA Long Beach Healthcare System, Long Beach, CA USA; 80 Minneapolis VA Health Care System, Minneapolis, MN USA; 81 Michael E. DeBakey VA Medical Center, Houston, TX USA; 82 Salem VA Medical Center, Salem, VA USA; 83 Manchester VA Medical Center, Manchester, NH USA; 84 St. Louis VA Health Care System, St. Louis, MO USA; 85 White River Junction VA Medical Center, White River Junction, VT USA; 86 VA Pacific Islands Health Care System, Honolulu, HI USA; 87 Syracuse VA Medical Center, Syracuse, NY USA; 88 Providence VA Medical Center, Providence, RI USA; 89 VA New York Harbor Healthcare System, New York, NY USA; 90 VA Pittsburgh Health Care System, Pittsburgh, PA USA; 91 North Florida/South Georgia Veterans Health System, Gainesville, FL USA; 92 VA Maine Healthcare System, Augusta, ME USA; 93 William S. Middleton Memorial Veterans Hospital, Madison, WI USA; 94 Overton Brooks VA Medical Center, Shreveport, LA USA; 95 New Mexico VA Health Care System, Albuquerque, NM USA; 96 VA Greater Los Angeles Health Care System, Los Angeles, CA USA; 97 Edith Nourse Rogers Memorial VA Hospital, Bedford, MA USA; 98 Clement J. Zablocki VA Medical Center, Milwaukee, WI USA; 99 San Francisco VA Health Care System, San Francisco, CA USA; 100 Atlanta VA Medical Center, Decatur, GA USA; 101 VA Western New York Healthcare System, Buffalo, NY USA; 102 Northport VA Medical Center, Northport, NY USA; 103 Raymond G. Murphy VA Medical Center, Albuquerque, NM USA; 104 Durham VA Medical Center, Durham, NC USA; 105 Canandaigua VA Medical Center, Canandaigua, NY USA; 106 Phoenix VA Health Care System, Phoenix, AZ USA; 107 South Texas Veterans Health Care System, San Antonio, TX USA

**Keywords:** Genome-wide association studies, Predictive markers, Non-alcoholic fatty liver disease

## Abstract

Serum concentration of hepatic enzymes are linked to liver dysfunction, metabolic and cardiovascular diseases. We perform genetic analysis on serum levels of alanine transaminase (ALT), alkaline phosphatase (ALP) and gamma-glutamyl transferase (GGT) using data on 437,438 UK Biobank participants. Replication in 315,572 individuals from European descent from the Million Veteran Program, Rotterdam Study and Lifeline study confirms 517 liver enzyme SNPs. Genetic risk score analysis using the identified SNPs is strongly associated with serum activity of liver enzymes in two independent European descent studies (The Airwave Health Monitoring study and the Northern Finland Birth Cohort 1966). Gene-set enrichment analysis using the identified SNPs highlights involvement in liver development and function, lipid metabolism, insulin resistance, and vascular formation. Mendelian randomization analysis shows association of liver enzyme variants with coronary heart disease and ischemic stroke. Genetic risk score for elevated serum activity of liver enzymes is associated with higher fat percentage of body, trunk, and liver and body mass index. Our study highlights the role of molecular pathways regulated by the liver in metabolic disorders and cardiovascular disease.

## Abstract (web summary)

Plasma levels of liver enzymes provide insights into hepatic function and related diseases. Here, the authors perform a genome-wide association study on three liver enzymes, identifying genetic variants associated with their plasma concentration as well as links to metabolic and cardiovascular diseases.

## Introduction

Global mortality due to liver disease has been on the rise since 2005^1^. Liver disease is now the third cause of premature mortality in the UK that kills 40 people a day in the UK alone overtaking deaths from diabetes and cancer^2^. While 90% of liver diseases can be prevented, 75% of the patients are diagnosed in late stages^2^. The great majority of liver disease in the UK is caused by alcohol consumption, obesity, and viral hepatitis, all of which may result in liver inflammation, cirrhosis, and hepatocellular carcinoma^2^.

Obesity is linked to liver disease through association with non-alcoholic fatty liver disease (NAFLD) or its newly proposed term metabolic (dysfunction)-associated fatty liver disease^3–5^. Research has shown an increased risk of cardiovascular disease (CVD) in people with NAFLD in both men and women^6^. Elevated serum activity of liver enzymes is an indicator of the underlying liver problems. Specific liver diseases such as NAFLD^2^, alcohol liver disease^7^, viral hepatitis^8^, autoimmune hepatitis^9^, and cholestatic disorders may have genetic underlying factors contributing to the initiation of liver disease or progression of the clinical course of the disease. Genetic factors are known to alter serum concentrations of liver enzymes^10^ and several genetic loci have been identified associated with serum activity of liver enzymes.

A previous genome-wide association study (GWAS) of serum activities of liver enzymes^11^ on ~60,000 individuals of European ancestry identified 44 genetic loci for serum level of alanine transaminase (ALT), alkaline phosphatase (ALP), and γ-glutamyl transferase (GGT).

Here, we sought to identify genetic factors involved in serum levels of ALT, ALP, and GGT using data from 437,438 individuals of European ancestry within the UK Biobank (UKB) and sought replication in 315,572 individuals of European ancestry from the Million Veteran Program (MVP), the Rotterdam Study, and the Lifelines Study. Our aim was to identify etiological genetic and molecular pathways underlying liver function and the link to metabolic disorders and CVDs. We identified and replicated the loci associated with serum activity of liver enzymes and highlighted the pathways involved in metabolic disorders and CVD.

We identified 517 liver enzyme single-nucleotide polymorphisms (SNPs) with evidence of involvement in liver development and function, lipid metabolism, insulin resistance, vascular formation, body mass index (BMI), and body and liver fat percentage. Liver enzyme SNPs show association with coronary heart disease and ischemic stroke.

## Results

**Fig. 1:**
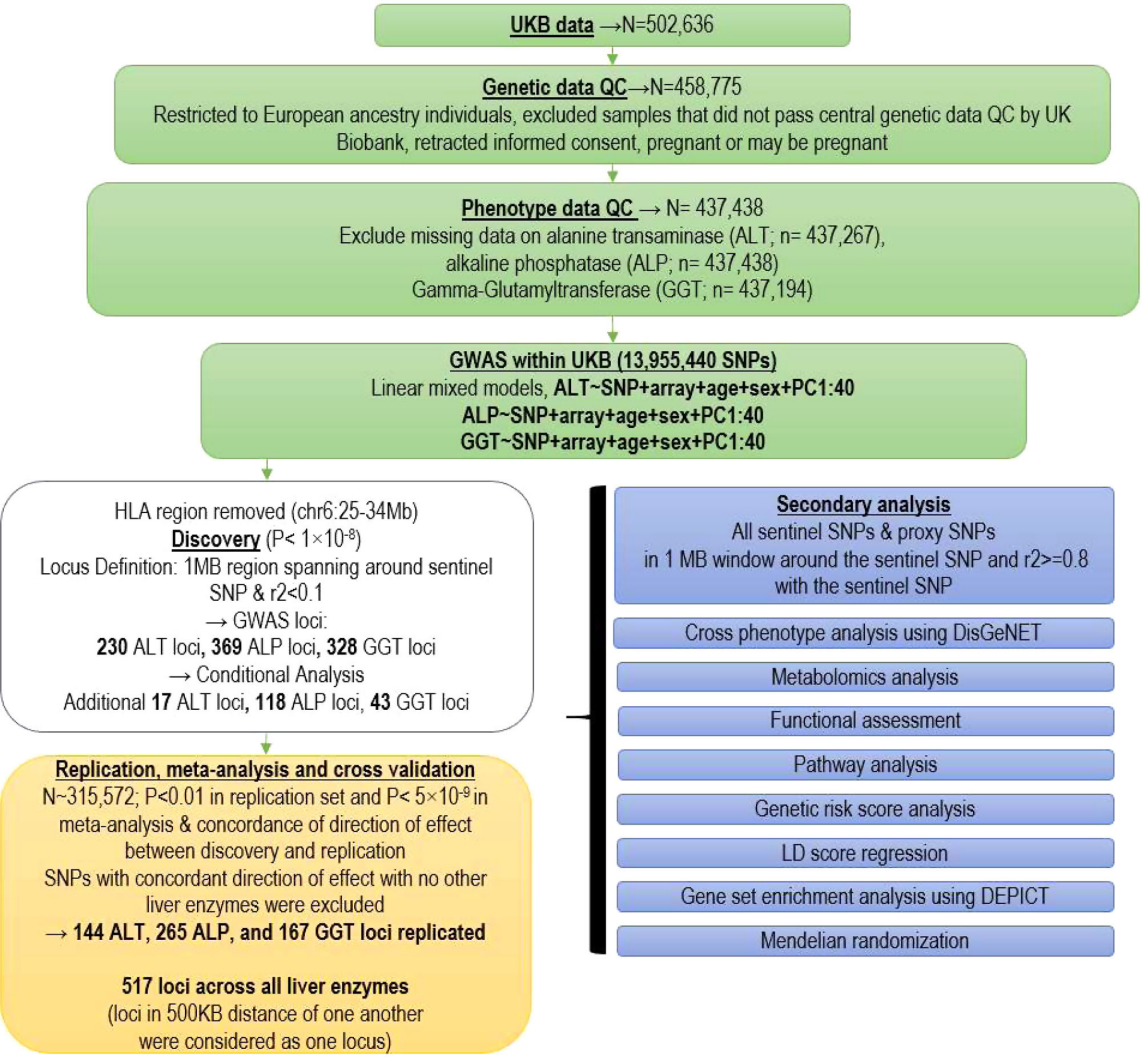
Overview of study design and findings. The figure illustrates the genotype and phenotype quality control (QC) within the UK Biobank (UKB) data. Statistical analysis and replication resulted in 517 loci associated with liver enzymes. PC principal component, SNP single-nucleotide polymorphism, GWAS-genome-wide association studies, LD linkage disequilibrium.

**Fig. 2:**
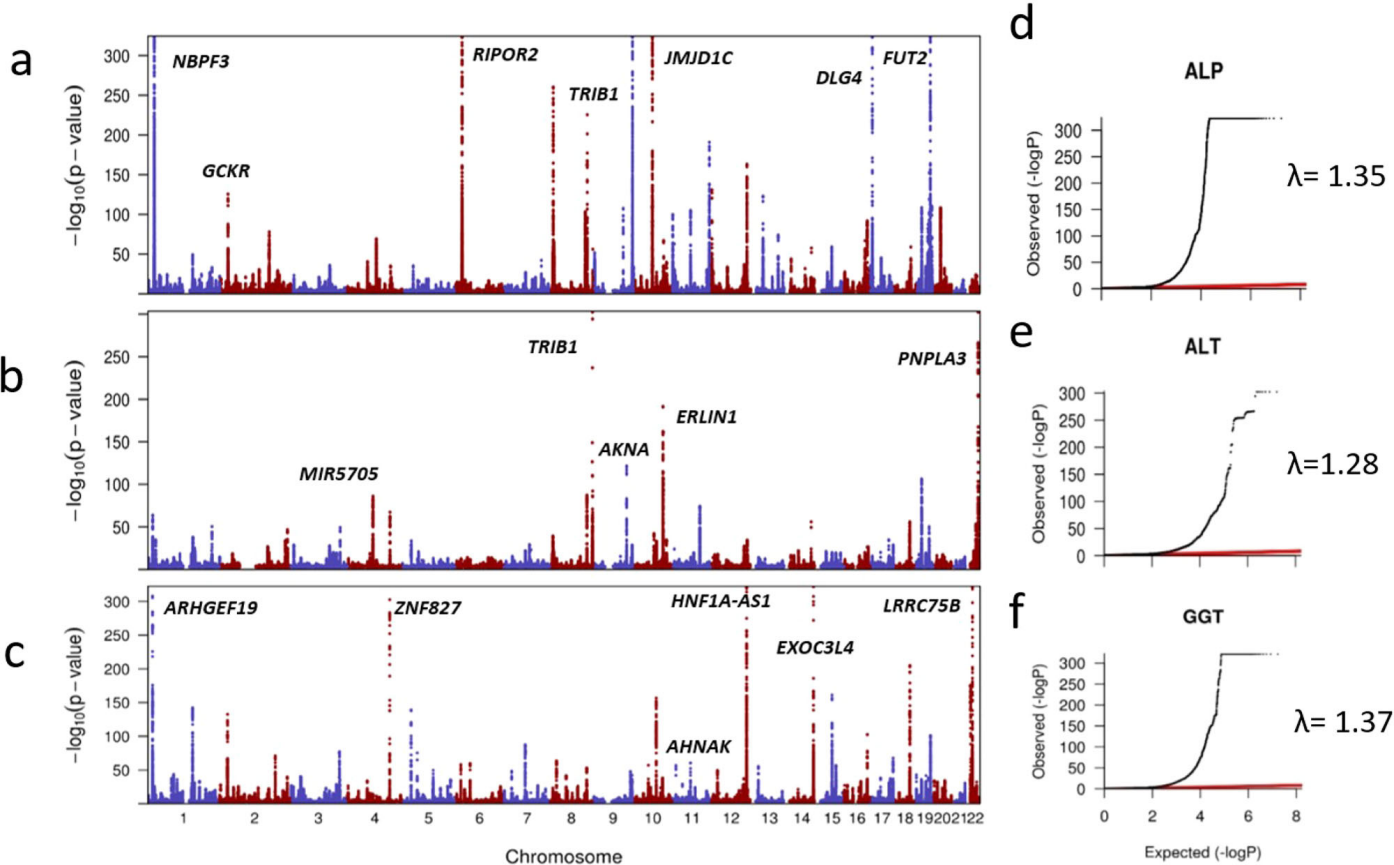
Overview of ALT, ALP, and GGT loci identified within the UKB study (discovery sample). Manhattan (MH) plots illustrated have been created based on summary statistics of GWAs on liver enzymes where the *x*-axis demonstrates chromosome number and the *y*-axis represents −log 10 (*P* value) for the association of SNP with liver enzymes. Q–Q plots are illustrated to show the inflation of test statistics using the summary statistics of the liver enzyme GWAS. Where the *x*-axis represents the expected log (*P* value). The red line shows the expected results under the null association. *Y*-axis illustrates the observed log (*P* value). **a** MH plot based on ALP GWAS summary statistics. **b** MH plot based on ALT GWAS summary statistics. **c** MH plot based on GGT GWAS summary statistics. **d** Q–Q plots for ALP, **e** Q–Q plots for ALT, and **f** Q–Q plots for GGT. Inflation of test statistics was represented by lambda (*λ*) values.

We performed a two-stage GWAS in European ancestry individuals on serum concentrations of ALT, ALP, and GGT using a discovery sample of 437,438 individuals (Fig. 1) and a replication sample of 315,572 individuals (Supplementary Data 1). At the discovery stage, Q–Q plots (Fig. 2) showed an early deviation from the expected line. To estimate if this is due to population stratification or polygenicity, we performed univariate linkage disequilibrium (LD) score regression (LDSR). The LDSR intercepts (standard error) in UKB were 1.12 (0.02) for ALT, 1.24 (0.02) for ALP, and 1.22 (0.02) for GGT, indicating that inflated test statistics are due to polygenicity of the traits. SNP heritability estimates (standard error) showed that 11% (0. 7%) of ALT, 20.9% (2%) of ALP, and 17% (1%) of GGT is heritable. At the discovery stage, we identified 328 SNPs for GGT, 230 for ALT, and 369 for ALP surpassing our pre-set stringent threshold at *P* < 1 × 10^−8^ (see “Methods”) within the UKB sample (Supplementary Data 2–4). Conditional analysis using the genome-wide complex traits analysis (GCTA) software^12^ identified additional independent SNPs for ALT (*n* = 17), ALP (*n* = 118), and GGT (*n* = 43).

**Fig. 3:**
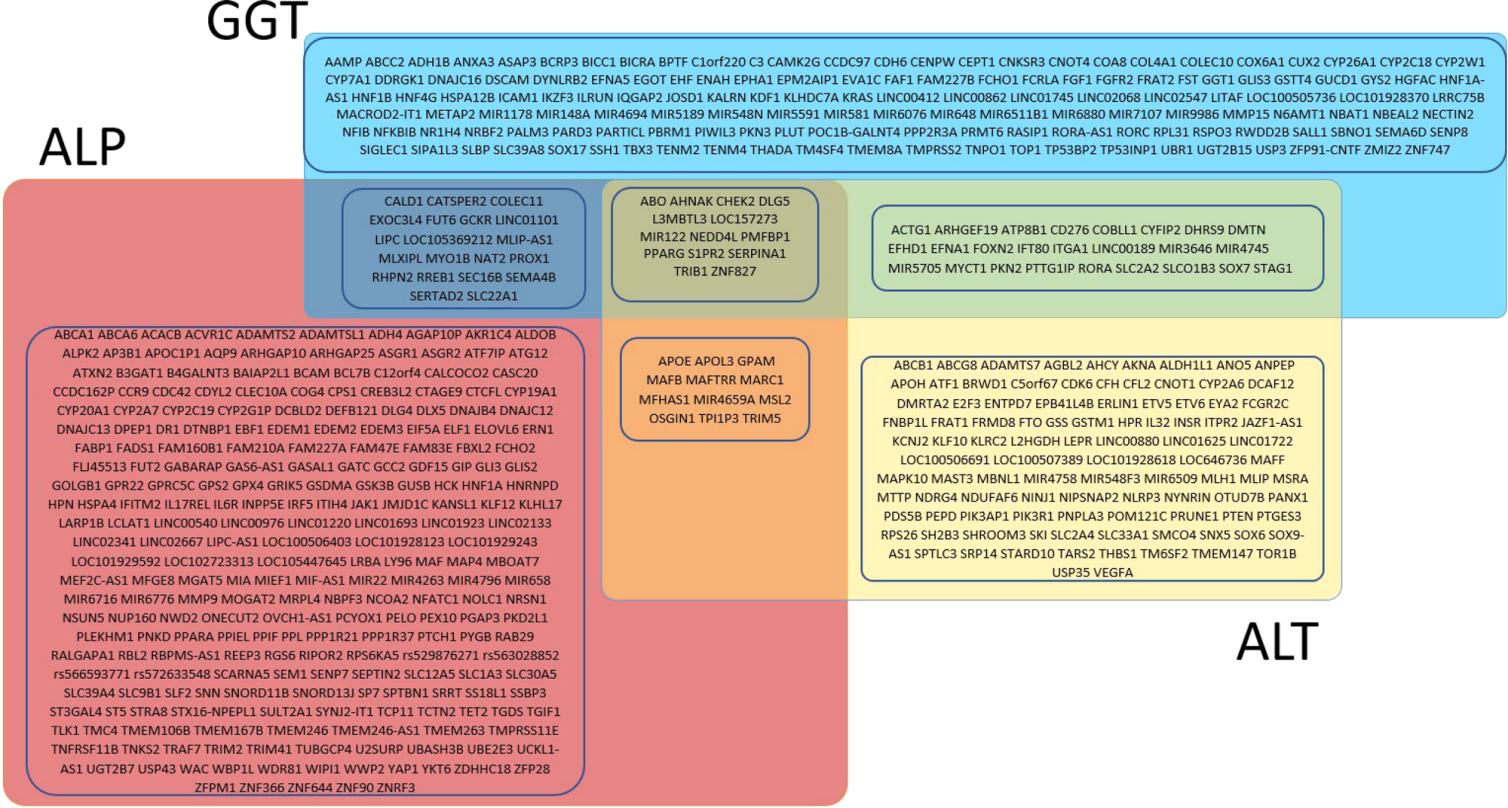
Overview of nearest genes mapped to known and novel ALT, ALP, and GGT replicated SNPs and their overlap. Yellow box depicts replicated genes mapped to ALT. Red box depicts replicated genes mapped to ALP. Blue box depicts replicated genes mapped to GGT. Boxes in overlapping sections depict genes identified to be associated with more than one liver enzyme.

We then sought replication of the discovered variants in three independent studies (total *N* = 315,572). We successfully replicated 517 SNPs including 144 ALT, 265 ALP, and 167 GGT SNPs (Fig. 3 and Supplementary Data 5–7) using our pre-set stringent replication criteria (see “Methods”).

We examined variance explained by the known and novel liver enzyme SNPs in the Airwave study^13^ cohort of UK police forces. We observed that ALT SNPs explained 10.3% variation in the circulating level of ALT; ALP SNPs explained 6.2% variation in the circulating level of ALP, and GGT SNPs explained 7.0% variation in the circulating level of GGT in the Airwave study.

### Cross-trait associations

To investigate evidence for shared genetic components with other traits, we used LDSR, which supports the hypothesis for shared genetic contribution with lipid and glucose metabolism, as well as coronary heart disease (CHD) across all three liver enzymes (Supplementary Fig. 1 and Supplementary Data 8). Liver enzyme SNPs showed positive genetic correlations surpassing our pre-set*P* value threshold of 1.94 × 10^−4^ with several cardiometabolic factors such as waist-to-hip ratio (*P*_ALT_ = 1.52 × 10^−55^; *P*_GGT_ = 1.19 × 10^−41^), type 2 diabetes (*P*_ALT_ = 1.77 × 10^−34^*P*_GGT_ = 1.16 × 10^−15^), CHD (*P*_GGT_ = 3.79 × 10^−23^; *P*_ALT_ = 2.17 × 10^−21^; *P*_ALP_ = 1.52 × 10^−8^), and high-density lipoprotein (HDL) cholesterol (*P*_ALT_ = 2.31 × 10^−13^). Meanwhile, liver enzyme SNPs showed negative genetic correlation with years of education (*P*_GGT_ = 1.13 × 10^−33^; *P*_ALT_ = 4.40 × 10^−29^; *P*_ALP_ = 6.45 × 10^−20^), parental age of first birth (*P*_ALT_ = 2.13 × 10^−21^; *P*_GGT_ = 3.36 × 10^−21^; *P*_ALP_ = 3.59 × 10^−10^), lung function (*P*_ALT_ = 2.18 × 10^−17^; *P*_GGT_ = 9.98 × 10^−11^; *P*_ALT_ = 5.67 × 10^−07^), and intelligence (*P*_GGT_ = 1.73 × 10^−10^; *P*_ALT_ = 1.73 × 10^−10^). Association of replicated liver enzyme SNPs with these genetically correlated traits are presented in Supplementary Data 9.

**Fig. 4:**
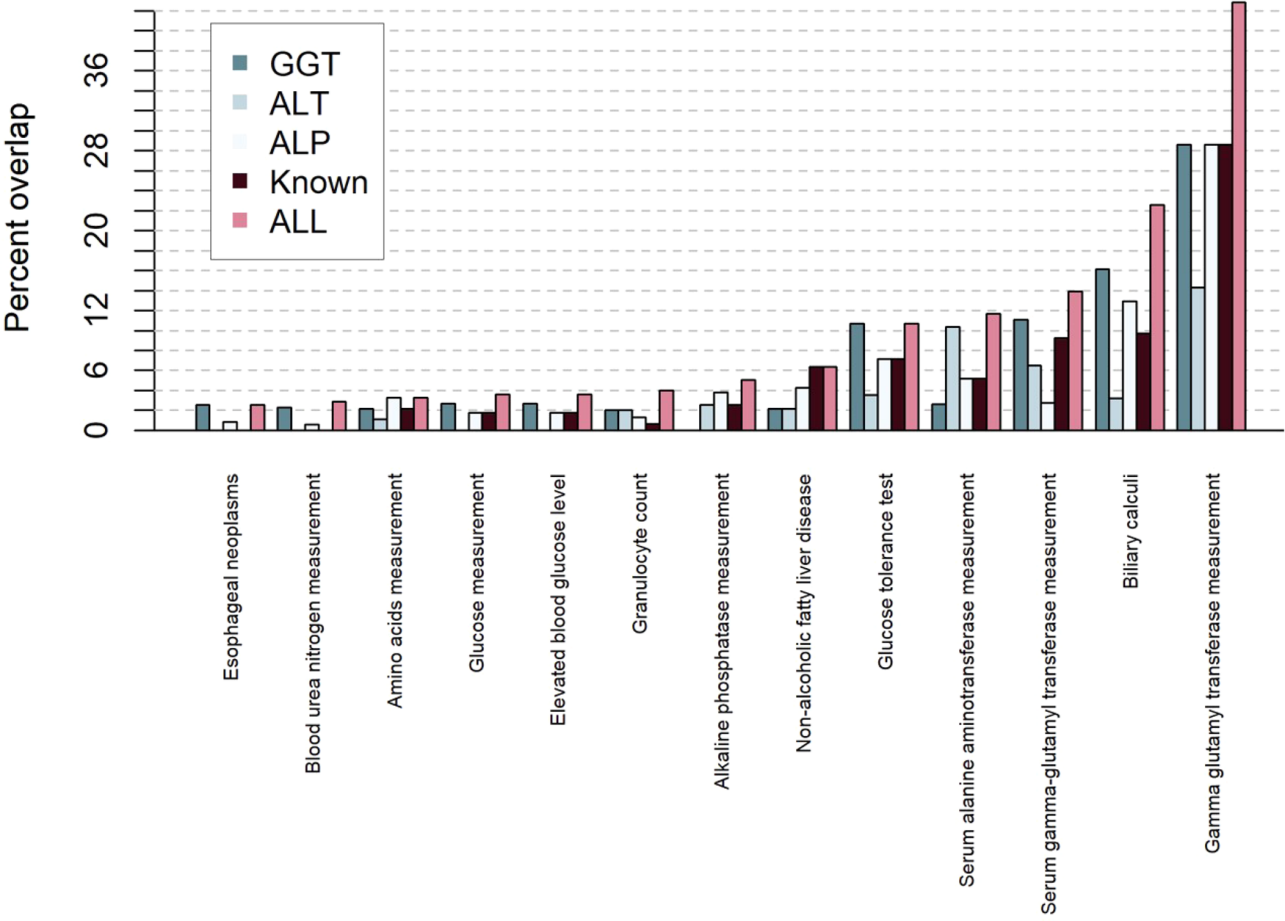
Overview of diseases and traits known to be related to liver enzyme SNPs using DisGeNET. Previous knowledge on the association of all (pink), known (brown), ALT (stone), ALP (light gray), and GGT(aegean) loci are depicted.

Assessment of cross-trait associations on DisGeNET^14,15^, a database on previously published gene–disease associations, showed that the ALT, ALP, and GGT known and novel SNPs were linked to multiple traits such as CVDs, lipid levels, alcohol consumption, NAFLD, and other cardiometabolic traits (Fig. 4). Metabolomics analysis showed that liver enzyme SNPs were mainly associated with lipid and drug metabolites (Supplementary Data 10).

### Tissue and protein expression assessment

We assessed gene expression of liver enzyme loci in 51 tissues (Supplementary Figs. 3–5). Genes mapped to liver enzyme genes showed medium to high gene expression in liver, adipose tissue, brain, artery, and urogenital system.

We compared the liver expression of genes mapped to our discovery stage SNPs with other tissues and we observed that among genes mapped to the identified SNPs, 26 ALP, 9 ALT, and 20 GGT SNPs were more expressed in the liver compared to all other 51 tissues. This result highlighted *SERPINA1* gene with the highest expression in the liver among all genes assessed. We also sought to identify which of the associated SNPs affect gene expression (expression quantitative trait locus (eQTL)) within the Genotype-Tissue Expression (GTeX) database. We found that 21 ALT, 31 ALP, and 30 GGT SNPs affected the expression of genes (*cis*-eQTL) across tissues. We then specifically looked for eQTL effects in the liver and observed that 5 ALT, 4 ALP, and 8 GGT SNPs (with one SNP overlapping between GGT and ALT) affected expression of genes in the liver (Supplementary Data 11). For example, ALP SNP rs5760119 (proxy SNP for rs5751777) had an eQTL effect on the expression of several genes in the liver including *DDT*, *DDTL*, *MIF*, and *GSTT2B*. Evaluation of protein expression information on the Human Protein Atlas^16^ available from www.proteinatlas.org showed high RNA and protein expression for *DDT*, *DDTL*, and *MIF* in the liver. We observed evidence of expression of a further ten liver enzyme genes *(SPTLC3*, *ACTG1*, *CD276*, *CHEK2*, *EFHD1*, *MIF*, *MLIP*, *NYNRIN*, *PGAP3*, and *SHROOM3*) in the liver or gallbladder.

### Pathway analysis

Using the Ingenuity Pathway Analysis (IPA)^17^ software, we found multiple canonical pathways involving gene lists mapped to the three liver enzyme SNPs. For example, the farnesoid X receptor (*FXR*) pathway that is involved in multiple biological systems including the metabolism of bile acid, lipids, glucose, and the immune system appeared as top canonical pathway across all three liver enzyme SNPs. Upstream regulator analysis identified multiple transcription regulators including nuclear receptors (*RXRA*, *NR1I2*, *ESR1*, *NR1H3*, and *PPARG*), and transcription regulators (*TP53*, *HNF4A*, *FOXA2*, and *CEBPA*).

**Fig. 5:**
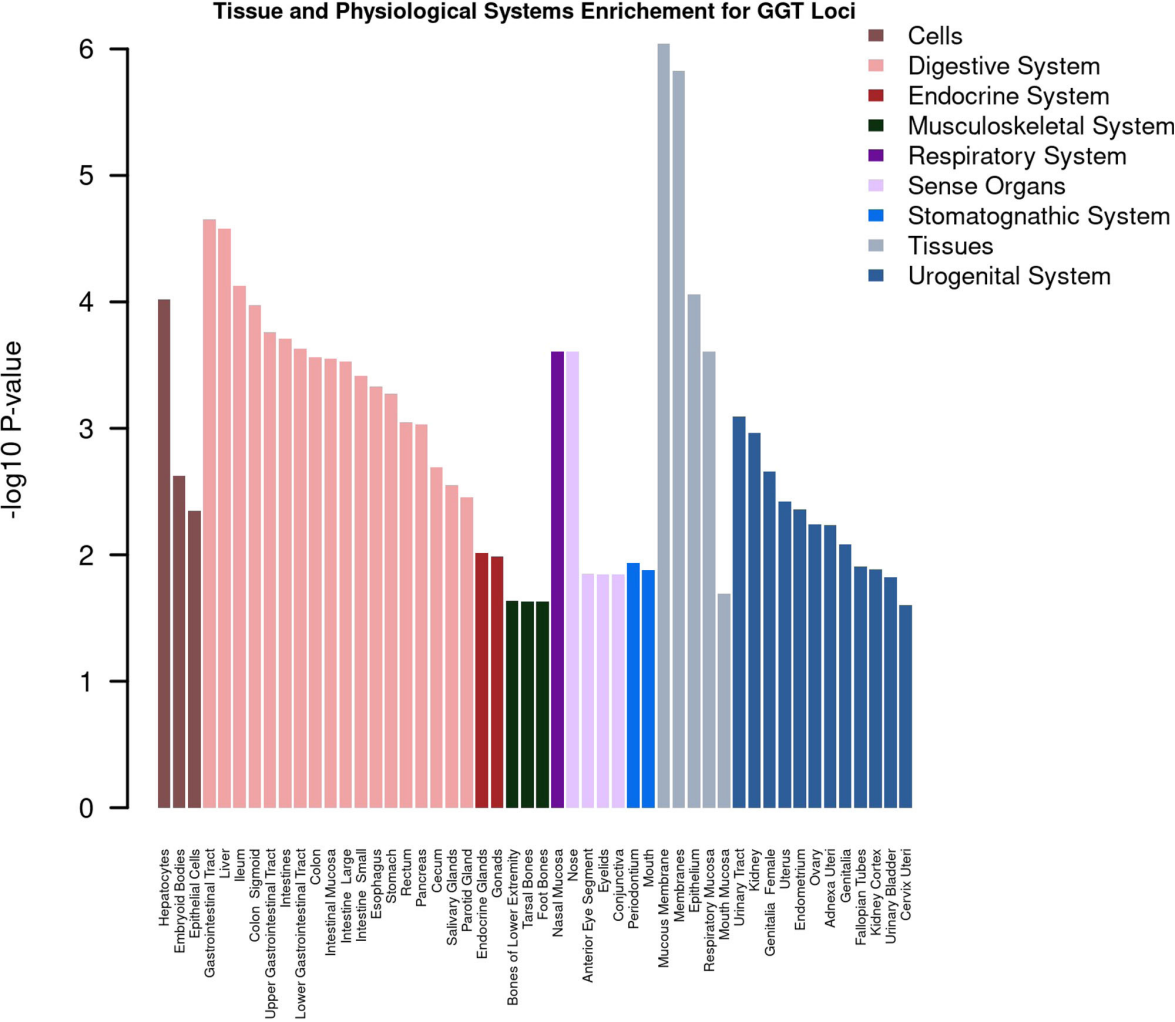
Overview of tissue enrichment for GGT SNPs using DEPICT. Illustrated are the tissues and organs enriched with genes mapped to GGT SNPs. False discovery rate <0.05 was used to identify enriched tissue/cells.

**Fig. 6:**
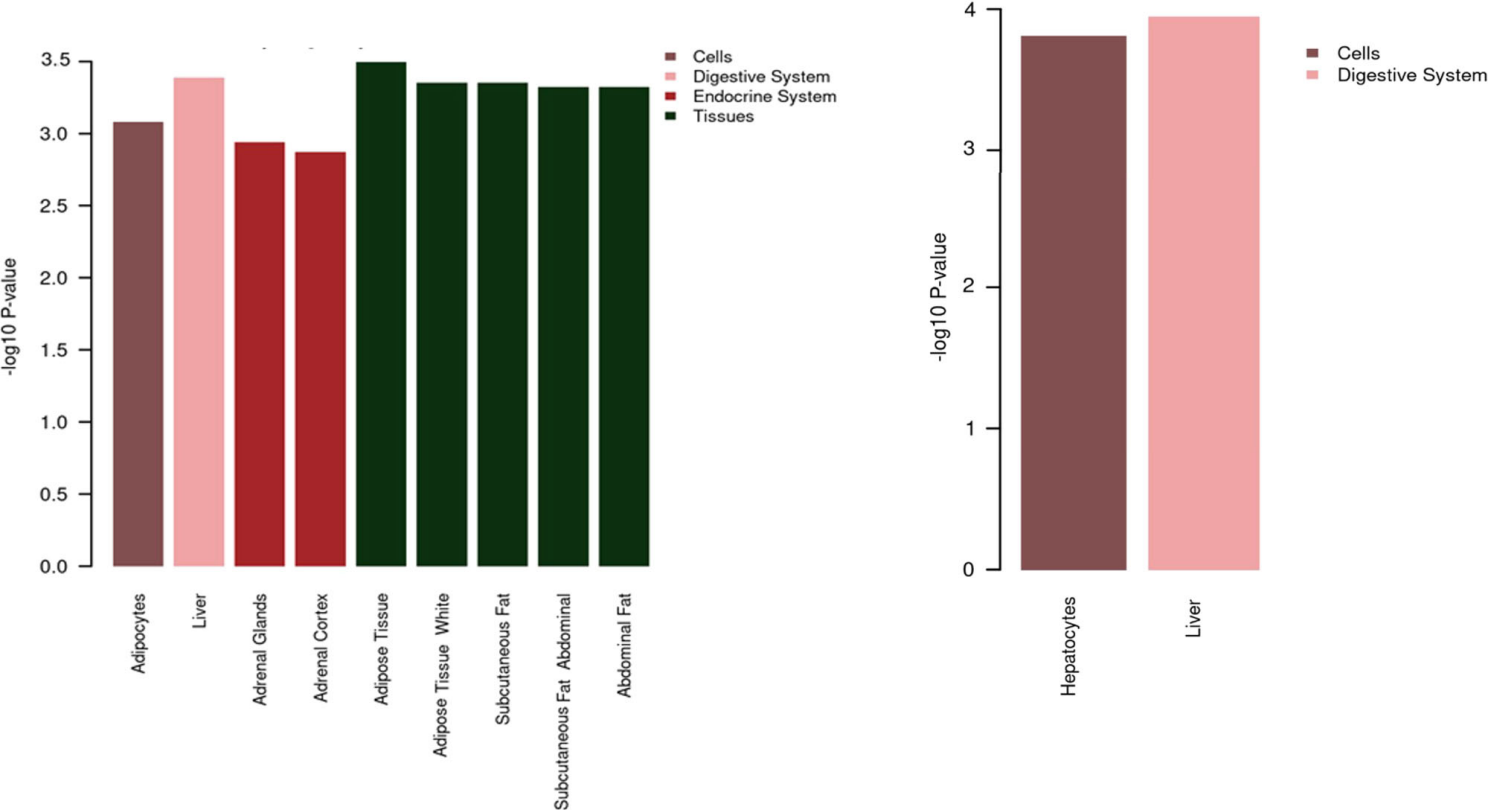
Overview of tissue and physiological systems enrichment using DEPICT. Illustrated are the tissues and organs enriched with genes mapped to ALT (**a**) and ALP (**b**) SNPs. False discovery rate <0.05 was used to identify enriched tissue/cells.

We also used Data-driven Expression Prioritized Integration for Complex Traits (DEPICT)^18^ to find gene sets associated with molecular pathways and tissues enriched with genes mapped to the liver enzyme SNPs. We identified enrichment across multiple organs, tissues, and cells (Figs. 5 and 6). We observed enrichment for ALT SNPs in the liver, adrenal glands, and adipocytes within a range of adipose tissues. ALP SNPs were enriched in hepatocytes in the liver and GGT SNPs were enriched mainly in hepatocytes, embryoid bodies, and epithelial cells across digestive, mucus membranes, and urogenital systems. Evaluation of enriched mammalian phenotypes in relation to liver enzyme SNPs highlighted the importance of a range of phenotypes including abnormal liver physiology and morphology, liver fibrosis, and abnormalities in lipid, glucose, bile acid, and iron metabolisms (Supplementary Data 12). Evaluation of Gene Ontology data in relation to all three liver enzyme SNPs showed the importance of retinoic acid receptor-binding pathway (*P* = 3.14 × 10^−7^), regulation of lipid biosynthetic process (*P* = 7.48 × 10^−7^), basolateral plasma membrane (*P* = 5.40 × 10^−9^), and multiple other pathways involved mainly in liver development and lipid homeostasis. Within KEGG and REACTOME pathways, we observed that enrichment of REACTOME *PPARA* activates the gene expression (*P* = 1.93 × 10^−9^) pathway, and regulation of lipid metabolism by *PPARA* gene expression activation (*P* = 2.86 × 10^−9^) were consistently enriched pathways across the three liver enzymes.

### Mendelian randomization (MR)

As our cross-trait assessment showed a link between liver enzyme loci with adiposity, lipid, and glucose metabolism that are the main risk factors for major cardiovascular events, we performed MR analysis to test the causality of the observed associations. To this end, we used the meta-analysis of discovery and replication samples to select the list of variants proxying liver enzyme levels, with genetic association estimates for CHD and stroke risk taken from previously published GWAS. We observed associations of genetically proxied serum levels of all three liver enzymes on CHD risk, although with heterogeneity in estimates obtained across methods that make different assumptions regarding the inclusion of pleiotropic variants. We also observed an MR association of ALT with ischemic stroke (Supplementary Data 13). MR using the inverse-variance-weighted (IVW) method showed that for 10-fold increase in genetically proxied serum level of ALT, the odds ratio (OR) for CHD was 5.84 (95% confidence interval (CI) = 2.52–13.52, *P* = 3.73 × 10^−5^). This was 2.15 (95% CI = 1.07–4.31, *P* = 0.03) per 10-fold increase in genetically proxied level of ALP and it was 1.46 (95% CI = 1.16–1.83, *P* = 0.001) per 10-fold increase in genetically proxied level of GGT. In addition, for 10-fold increase in genetically proxied ALT, the OR for ischemic stroke was 2.33 (95% CI = 1.30–4.19, *P* = 0.005).

### Genetic risk score (GRS) analysis

To investigate cumulative effect of liver enzyme SNPs on various complex traits, we performed GRS analysis in the Airwave sample. The GRS was weighted according to the meta-analysis effect estimates for serum level of liver enzyme SNPs (Supplementary Tables 5–7). Here, each standard deviation of increase in ALT GRS was associated with 3.09 U/L in ALT (95% CI = 2.02–4.17; *P* = 3.5 × 10^−8^). Each standard deviation increase in ALP GRS was associated with 2.07 U/L in ALT (95% CI = 1.49–2.66; *P* = 3.05 × 10^−11^), whereas each standard deviation increase in GGT GRS was associated with 1.43 U/L increase in GGT (95% CI = 1.35–1.52; *P* = 2.58 × 10^−210^). We similarly observed association between GRSs and liver enzymes in NFBC1966 cohort for serum levels of ALT (OR = 1.72; 95% CI = 1.36–2.07; *P* = 7.55 × 10^−21^), ALP (OR = 1.88; 95% CI = 1.67–2.09; *P* = 1.32 × 10^−65^), and GGT (OR = 1.96; 95% CI = 1.72–2.19; *P* = 2.98 × 10^−56^).

We investigated the association of GRS with liver and metabolic traits (see “Methods”) within UKB (Supplementary Data 14). GRS was associated with the metabolic syndrome (*β* = 0.001; 95% CI = 0.001–0.01; *P* = 2.47 × 10^−38^), and body fat distribution indices such as body fat percent (*β* = 0.07; 95% CI = 0.05–0.09; *P* = 5.97 × 10^−13^), and liver fat percent (*β* = 0.28; 95% CI = 0.13–0.42; *P* = 1.28 × 10^−4^). Our liver enzyme GRS showed a marginal inverse association with basal metabolic rate (*β* = −2.76; 95% CI = −5.3 to −0.23; *P* = 0.03) and left ventricular diastolic volume (*β* = −1.77; 95% CI = −3.51 to −0.03; *P* = 0.04). We additionally observed that liver enzyme GRS was associated with a small increase in the risk of incident CVD (OR = 1.03; 95% CI = 1.01–1.05; *P* = 6.47 × 10^−4^). To investigate the mediatory/confounding effect of adiposity, lipid, and glucose metabolism on the association of GRS and CVD, we corrected our CVD analysis for the effect of body fat percent, BMI, and the metabolic syndrome, as well as biomarkers of lipid and glucose metabolism. Of these factors, we observed that adjustment for metabolic syndrome or HDL cholesterol gave a partial reduction in risk of liver enzyme GRS on CVD (Supplementary Data 15) more than other factors.

## Discussion

We performed a GWAS for serum activity of liver enzymes using a sample size of 437,438 participants from the UKB study and replicated the findings among 315,572 individuals from three independent cohorts of European ancestry, in a combined sample size of 753,010. Using this design, we identified 517 SNPs associated with the serum level of three liver enzymes. These SNPs explained 6–10% of the variation in the liver enzyme levels in an independent study. Our analysis indicates an SNP-based heritability of 11% for ALT, 17% for GGT, and 21% for ALP. These estimates are much higher (up to 10%) than previously reported SNP-based heritability estimates for serum activity of liver enzymes^19^.

Genetic correlation analysis supports that genetic determinants of liver enzyme serum levels are linked to lipid and glucose metabolism, adiposity, and CVDs. Metabolomics analysis highlighted the association of lipids and lipoproteins with individual liver enzyme loci. We additionally showed that liver enzyme SNPs collectively are associated with increased lipid levels, increased body fat distribution indices, increased insulin-like growth factor-1 and hemoglobin A1C, and increased NAFLD. In GRS association with CVD, we showed that adjustment for metabolic syndrome or HDL gave 10–15% reduction in the effect size of liver enzyme GRS on CVD, implying that some of this CVD risk may be attributable to the metabolic syndrome/ lipid metabolism.

The top canonical pathway analysis by IPA highlighted the role of FXR, a nuclear receptor involved in the regulation of bile acid synthesis and transport^20^. The FXR pathway is known to protect against liver inflammation associated with non-alcoholic steatohepatitis^21^ and is involved in lipid transport and glucose metabolism. The biological links within the FXR pathway may provide a biological support for the observed link between liver enzyme loci, lipid dysregulation, diabetes, and obesity.

Furthermore, our gene-set enrichment analysis using DEPICT^18^ once again highlighted the regulation of lipid metabolism processes and abnormal liver physiology and morphology. These in silico analyses from multiple sources suggest interconnectivity of lipid and glucose metabolism with processes involved in liver physiology and morphology.

Among the genes identified, we found that *LIPC* (hepatic type of lipase C) is associated with liver enzyme levels. This gene is highly expressed in the liver and is involved in receptor-mediated lipoprotein uptake, affecting lipid levels^22^. Polymorphisms in *LIPC* have been associated with hypertension, type 2 diabetes, and metabolic syndrome^23^. Since familial lipid disorders such as familial combined hyperlipidemia^24^ that commence in infancy are known to cause NAFLD, changes in lipid levels due to polymorphisms in genes such as *LIPC* might occur prior to changes in serum activity of liver enzymes, perhaps due to the accumulation of fat in the liver. This also applies to another liver enzyme locus, *APOE*, that is a well-studied lipid-modulating locus linked to *LIPC* and hepatic injury.

We observed a genetic correlation between femoral neck bone mineral density and ALP in our discovery stage within the UKB. This was not the case for ALT or GGT. ALP has multiple isoforms with the bone and liver being the most abundant circulating isoforms^25^. In our replication strategy, for each locus to be considered replicated, we implemented concordance of effect with another liver enzyme. This strategy filtered out the signals that were probably due to bone diseases rather than the liver and eventually none of the replicated ALP SNPs reported here show the previous link to bone traits.

Our study additionally confirms the association of various loci that have been shown to be involved in liver disorders. A recent GWAS on non-alcoholic fatty liver and steatohepatitis by Anstee et al.^26^ highlighted the role of *PNPLA3*, *TM6SF2*, *GCKR*, *PYGO1*, *HSD17B13*, and *LEPR* in these liver disorders. In addition, a recent GWAS on NAFLD by Namjou et al.^27^ highlighted the role of *TRIB1*, *PNPLA3*, *TM6SF2*, *COL13A1*, and *GCKR* in the pathogenesis of NAFLD. Our study confirms that SNPs in *PNPLA3*, *TM6SF2*, *GCKR*, and *LEPR* are associated with the serum activity of liver enzymes.

Some of the SNPs we replicated play a role in rare familial liver disorders. For instance, we identified and replicated SNPs in *SLC22A1*, *LIPC*, *ABCC2*, *CYP7A1*, *NR1H4*, *ADH4*, *MTTP*, and *ATP8B1* regions that have been previously linked to familial intrahepatic cholestasis^28^. The disease onset is in childhood and manifests with cholestasis in the liver, leading to liver failure. The pathologic underlying factors are defects in bile acid secretion and metabolism.

One of our lead SNPs in *SERPINA1* gene rs28929474 has previously been associated with liver traits, and mutations in *SERPINA1* is known to cause liver cirrhosis^29^. Our study confirms a strong association between this locus across all three liver enzymes.

In summary, here we increase the number of SNPs identified so far for modulating circulating liver enzymes to a total of 561 SNPs. Our tissue expression lookup supported the role of genes with strong evidence of expression in the liver or gallbladder. We show evidence of involvement of liver enzyme SNPs in metabolic syndrome and in coronary artery disease. Our study shows that up to 10% of the variance in serum activity of liver enzymes is genetically determined and suggests the possible role of SNPs involved in liver fat percent in variation in serum activity of liver enzymes and a shared genetic contribution with CVD. Our study implies a role for genetic loci for liver enzyme levels in creating multiple abnormalities in lipid, glucose, and bile acid metabolism. These disturbances seem to be linked to the accumulation of fat in the liver and the body, as well as abnormalities in lipid levels, glucose control, and liver enzyme levels. Adiposity, hyperlipidemia, and abnormal glucose metabolisms are known to be linked to accelerated atherosclerosis and CVD risk. Dedicated investigations are needed on the biological effect of genes within the FXR pathway, their physical interaction, and their link to liver abnormalities and cardiometabolic changes.

## Methods

### Study design and participants

We used data from the UKB^30–32^ and included 437,267 individuals aged 40–69 years in the discovery stage. Study participants were ascertained through United Kingdom National Health Service registers across 22 centers in Great Britain between 2006 and 2010^32^. We included individuals of European ancestry following quality measures and exclusions (sex discordance, high missingness, and/or heterozygosity). Allocating individuals to ethnicity groups was based on self-reported ethnicity matched with principal component analysis ancestry clustering using the *k-means* clustering method. We excluded participants who had withdrawn consent (*n* = 39), as well as those who were pregnant or unsure of their pregnancy status at recruitment (*n* = 372). Non-European ancestry individuals were excluded from the main analysis. We limited our analysis to individuals with complete values for ALT, ALP, and GGT concentration. After exclusions, there were 437,267 individuals for ALT analysis, 437,438 for ALP, and 437,194 for GGT (Fig. 1) analyses. Values of ALT, ALP, and GGT were log 10 transformed to approximate normal distribution. To replicate our SNPs, we used data for 315,572 individuals from three independent studies, namely (i) the Rotterdam Study (NL, *N* = 6943)^33^; (ii) the Lifelines study (NL, *N* = 13,386)^34^; and (iii) the MVP (USA, *N* = 294,043)^35^ (see Supplementary information). For additional replication, we used GRS and sought the effect estimate and explained variance of the GRS on serum level of ALT, ALP, and GGT in independent samples from the Airwave health monitoring study^13^, a cohort of UK police forces, and in the Northern Finland Birth Cohort 1966^36,37^ (NFBC1966; see Supplementary information).

For subsequent analyses such as the association of GRS with various trait and association testing with NAFLD within the UKB, we excluded 918 individuals who had (based on Hospital Episode Statistics [HES] at the time of recruitment) documented International Classification of Diseases Tenth Revision (ICD10) diagnosis code for osteopathy (M45-49 and M80-90), vitamin D deficiency (E55), any liver disorders (K70-K77) including NAFLD (ICD10 code K760), alcohol liver disorder (K70), primary biliary cholangitis (PBC; K74.3), primary sclerosing cholangitis (PSC and K83), autoimmune hepatitis (AIH; K75.4), diseases of the gallbladder (K80-K87), and parathyroid diseases (E214, E215, D351, C750, and D442).

### Ethical consideration

The North West Multi-Center Research Ethics Committee has approved the UKB study. Any UKB participants who withdrew consent were removed from the current analysis. Local ethical approval was obtained for all independent replication cohorts.

The MVP received ethical and study protocol approval from the Veteran Affair Central Institutional Review Board and site-specific Research and Development Committees in accordance with the principles outlined in the Declaration of Helsinki. Informed consent was obtained from all participants of the MVP study.

Lifelines are conducted according to the principles of the Declaration of Helsinki and is approved by the medical ethics committee of the University Medical Centre Groningen, The Netherlands. Written informed consent was obtained from all participants.

The Rotterdam Study has been approved by the medical ethics committee according to the Population Screening Act: Rotterdam Study, executed by the Ministry of Health, Welfare, and Sports of the Netherlands. All participants from the Rotterdam Study in the present analysis provided written informed consent to participate and to obtain information from their treating physicians.

The Airwave Health Monitoring Study is approved by the National Health Service Multi-site Research Ethics Committee (MREC/13/NW/0588).

The NFBC1966 study was approved by the Ethics Committee of the Northern Ostrobothnia Hospital District, and the Ethics Committee of the University of Oulu. All participants gave written informed consent.

### Liver and metabolic traits

The serum concentration of ALT, ALP, and GGT in stored blood samples was measured using the enzymatic rate analytical method on a Beckman Coulter AU5800. The manufacturer’s analytic range for ALT was 3–500 U/L, for ALP, 5–1500 U/L, and it was 5–1200 U/L for GGT. Details of quality control and sample preparation for the measurements of serum activity of liver enzymes have been published by the UKB^38^.

We investigated the effect of genetic determinants of liver enzyme levels on BMI, basal metabolic rate (explain methods), electrocardiographic traits, left ventricular ejection fraction, cardiac index, bioimpedance measures using the Tanita BC418MA body composition analyzer including basal metabolic rate, body fat mass, body fat percentage (*n* = 415,692), fat-free mass, predicted muscle mass, and impedance for the trunk (*n* = 415,667), as well as coronary artery disease. Liver fat distribution was available in a subset of the UK Biobank, which had undergone imaging analysis of the liver and had genetic data available (*n* = 4085).

### Cardiovascular events

UK Biobank data are linked to electronic health data including HES and Office for National Statistics cause of death data. HES data provide information on hospital admissions for diagnoses and procedures. Using HES we defined CVD as coronary artery disease, stroke, or myocardial infarction classified using our published algorithm^39^ comprising codes from the ICD 9th (428, 410, 411, 412, 413, 414, 4297, 431, 430, 434, 436, 428, 425) and 10^th^ (I20, I21, I22, I23, I24, I25, I61, I60, I63, I64, I61, I60, I50, and I42) Revision codes. Prevalent cases were removed from the analyses.

We additionally investigated electrocardiographic traits, left ventricular ejection fraction, and cardiac index in relation to genes identified in this study.

### Genotyping and Imputation

Genotyping and imputation in the UKB have been described in detail elsewhere^40,41^. Briefly, two custom Affymetrix UKBileve and UKB Axiom arrays^42^ (designed to optimize imputation performance) were used for genotyping of DNA samples obtained from the UKB study participants. The UKB performed imputation centrally using an algorithm implemented in the IMPUTE2 program. Only markers that were present in both UKBileve and UKB Axiom arrays were used for imputation. To maximize the use of haplotypes with British and European ancestry, a special reference panel comprising a merged sample of UK10K sequencing and 1000 Genomes imputation reference panels was used for genotype imputation by the UKB. Genetic principal components to account for population stratification were computed centrally by UKB.

### Genome-wide association analysis in UKB

We restricted the main association analysis to SNPs from the third release of UKB genetic data (GRCh37). For GWAS on serum activity of liver enzymes, we performed linear mixed models (LMM) as implemented in the BOLT-LMM (v2.3) software^43^. The BOLT method accounts for the population structure and cryptic relatedness simultaneously. We assumed an additive genetic model on log 10-transformed ALT, ALP, and GGT values, adjusted for age, sex, and 40 genetic principal components for European ancestry. We applied several filters on a random subset of individuals and common SNPs (minor allele frequency [MAF] > 5%) to estimate parameters of LMM with Hardy–Weinberg equilibrium *P* > 1 × 10^−6^ and missingness <0.015 for the initial modeling step.

For the BOLT-LMM analysis to estimate the effect of SNPs on serum level liver enzymes, we set the discovery stage significance threshold of *P* < 1 × 10^−8^. This stringent threshold (compared with the usual GWAS threshold of *P* < 5 × 10^−8^) was used to robustly define lead SNPs to be put forward for replication and functional assessment. Multiallelic SNPs were removed from the database. We removed all SNPs in the HLA region (chr6:25-34 MB) and removed SNPs with MAF < 0.001. A total of 13,995,440 SNPs passed our quality control criteria and were included in ALP, ALT, and GGT GWAS.

Genetic data of the UKB include many SNPs in high LD that might inflate GWAS test statistics. To distinguish confounding due to population stratification from polygenicity in such data, we applied a univariate LDSR method^44^. We calculated LDSR intercept for ALP, ALT, and GGT GWAS, which was then used as a genomic control factor to account for cryptic relatedness.

### Locus definition

For the selection of lead SNPs at the discovery stage, all associations surpassing the stringent threshold of *P* < 1 × 10^−8^ were ranked in order of statistical significance with the strongest SNP associations located at the top of the list. We then removed all SNPs in the region of ±500 kb spanning the strongest ranking SNPs (lead SNP) that showed larger association *P* values than the lead SNP. We additionally LD pruned the list of final lead SNPs considering SNPs with LD threshold of *r*^*2*^ < 0.1 as independent signals.

To detect any secondary signals, we used UKB GWAS summary-level data for ALT, ALP, and GGT and performed approximate conditional analysis using the GCTA software^12^. We used locus-specific conditional analysis for ALT, ALP, and GGT conditioned on the lead SNPs within each locus. Our criteria for the selection of secondary signals included MAF ≥ 0.001 and *P* < 1 × 10^−8^ both in the BOLT-LMM GWAS and in joint conditional analysis within GCTA. The individual-level data for the European ancestry participants of UKB were used for LD calculation in GCTA analysis. We accepted and added the signals passing these selection criteria to the list of lead SNPs.

For further exploratory analyses, we searched proxy SNPs (*r*^2^ > 0.8) within 1 Mb region spanning the final LD pruned lead SNPs. Our criteria to choose proxy SNPs included location within 1 Mb window around the sentinel SNP and *r*^2^ ≥ 0.8 with the sentinel SNP. For proxy SNPs to be eligible for further analyses, we used MAF ≥ 0.001 and an imputation score >0.3. Both LD pruning and proxy search were performed using the PLINK2 software^45,46^.

### Replication and concordance

We sought replication for all independent lead SNPs from the BOLT-LMM and GCTA analysis in independent samples. We used data from multiple cohorts of (i) the Rotterdam Study (*n* = 6943)^33^, (ii) the Lifelines study (*n* = 13,386)^34^, (iii) and the Million Veterans Program (*n* = 294,043)^35^, and performed a meta-analysis across all replication cohorts. Later, we carried out a meta-analysis of discovery and replication results using inverse-variance fixed-effects models in the METAL software^47^. Our replication criteria included (i) stringent (*P* < 5 × 10^−9^) association *P* value in the meta-analysis of discovery and replication, to minimize false-positive signals; (ii) *P* < 0.01 in the meta-analysis of replication cohorts together with the concordant direction of effects in the meta-analysis of replication and discovery; (iii) concordant direction of effects on serum level of at least two of the three liver enzymes. In addition, we cross-referenced the ALP-replicated SNPs against reports of bone traits reported in GWAS Catalog^48^ to exclude any potential bone signals. We listed all unique replicated SNPs across all three liver enzymes, and we considered every two SNPs in 500 kb distance of one another as a single locus.

### Cross-trait associations

In addition to the final replicated SNPs, we included their proxy SNPs (*r*^2^ ≥ 0.8) for functional assessment and cross-trait lookups.

To investigate shared heritable contribution between serum activity of liver enzymes and other phenotypes, we used the Broad institute LD hub^49^ tool on 257 LD hub traits (excluding Neal’s lab GWAS analyses http://www.nealelab.is/uk-biobank/ that are based on UKB) to agnostically assess the genetic correlation between any two given traits using LDSR method^44^ implemented in online LD hub tool. The LDSR method developed by Bulik-Sullivan lab uses summary statistics from previously published GWAs. The method estimates genome-wide genetic correlation calculated from the additive genetic variance and covariance between any pair of traits^44^. We used three GWAS summary statistics data from our discovery stage for ALP, ALT, and GGT traits against 257 LD hub summary statistics creating 771 combinations of paired traits. LDSR method uses summary statistics from GWAs of two different traits to identify the genetic correlation between the two traits using SNP data and is described in detail by Bulik-Sullivan et al.^44^. To claim significance, we used a *P* value threshold of 1.94 × 10^−4^ corresponding to a nominal *P* value (0.05) with Bonferroni correction for 257 LD hub traits.

To assess and identify disease traits that are linked to ALT, ALP, and GGT SNPs, we sought evidence of previous associations using DisGeNET^14,15^. As input, we used ALT, ALP, and GGT lead SNPs and their proxy SNPs (*r*^2^ > 0.8) within 1 Mb region.

To investigate the metabolomic signatures of the identified SNPs, we used individual-level metabolomics data on 1941 serum samples from the Metabolon platform in the Airwave study^13^, a cohort of UK police forces, and performed association tests using linear regression analyses, adjusted for age and sex and principal components of genetically inferred ancestry.

### Tissue and Protein expression analysis

We used the online portal of the GTEx database^50–52^ to obtain the multi-tissue eQTL summary statistics (V7) on gene expression levels by Transcripts per Million using expression data from 48 tissues. To account for multiple testing, we used Benjamini–Hochberg corrected *P* values to denote statistical significance.

We additionally retrieved median gene expression levels by Transcripts per Million for genes mapped to ALT, ALP, and GGT SNPs from the RNA seq GTEX (V7) database for 51 tissues. For each tissue, we calculated mean and standard deviations of gene expression values. We then standardized gene expression levels across gene transcript-tissue combinations from GTEx to facilitate comparison across tissues. We finally used proteomics (https://www.proteomicsdb.org), tissue expression databases (https://tissues.jensenlab.org), and human protein atlas^16^ (www.proteinatlas.org) to check for protein expression of the genes in eQTL with liver enzyme SNPs.

### Pathway analysis and gene-set enrichment analysis

We annotated replicated SNPs to the nearest gene within a distance of ±500 kb using the University of California Santa Cruz (UCSC) genome browser. We performed gene-based variant effect analysis using the IPA^17^ software (IPA®, Qiagen Redwood City) on genes mapped to ALT, ALP, and GGT SNPs to evaluate over-representation of these genes in canonical pathways and in association with previously reported diseases and biological functions.

The *P* value of overlap implemented in IPA states the statistical significance of the enrichment of a biological attribute (e.g., canonical pathway, upstream analysis, etc.) in the user’s dataset. It compares the proportion of input molecules (e.g., genes) that are associated with a particular biological attribute to the proportion of molecules that we expect to see if the dataset were made up of randomly selected molecules. It is calculated using the right-tailed Fisher’s exact test. A *P* value < 0.05 or (−log *P* value = 1.3) is considered significant by IPA. The smaller the *P* value, the less likely that the association is random and the more statistically significant the association^53^.

For our replicated SNPs for each of the three liver enzymes, we used DEPICT^18^ at enrichment false discovery rate <0.05 to highlight gene sets associated with specific molecular pathways and mammalian phenotypes.

### GRS analysis

To estimate the cumulative contribution of genetic variants to liver enzyme concentrations, we created a GRS for the novel and known loci, weighted according to the effect estimates from the meta-analysis of discovery and replication (*n* = 753,010). This was separately done across all three liver enzyme SNPs and then an average value of the three GRSs was calculated. This averaged GRS was then standardized so that each unit in the GRS represents 1 SD. We tested the GRS against liver enzyme levels in the independent Airwave study (*n*_ALP_ = 331; *n*_ALT_ = 330; *n*_GGT_ = 13,420)^13^ and estimated the percentage of variance in serum activity of liver enzymes explained by the GRS. We additionally replicated the GRS results in the NFBC1966 cohort (*n*_ALP_ = 3619; *n*_ALT_ = 3620; *n*_GGT_ = 3617).

To test the involvement of replicated liver enzyme SNPs in complex conditions and diseases relevant to the liver, we created a GRS within the UKB weighted according to effect estimates from the meta-analysis of independent replication cohorts (*n* = 315,572). We investigated the association of this GRS with liver and metabolic traits (described above) within UKB.

### Mendelian randomization

To further investigate the effect of circulating levels of the liver enzymes on the risk of cardiovascular outcomes, a two-sample MR approach was employed^54^. We considered the outcomes of CHD, ischemic stroke, and intracerebral hemorrhage (ICH). Genetic association estimates on outcomes were obtained from the CARDIoGRAMplusC4D Consortium for CHD (60,801 cases and 123,504 controls, multiethnic)^55^, the MEGASTROKE Consortium for ischemic stroke (60,341 cases and 454,450 controls, multiethnic)^56^, and the International Stroke Genetic Consortium for ICH (1545 cases and 1481 controls, European ancestry)^57^. For the main analysis, the random-effects IVW meta-analysis MR approach was used, with the simple and weighted median, and MR-Egger approaches also employed as sensitivity analyses as these are more robust to the inclusion of potentially pleiotropic variants^58^.

### Reporting summary

Further information on research design is available in the Nature Research Reporting Summary linked to this article.

## Supplementary information

Supplementary Information

Description of Additional Supplementary Files

Supplementary Data

Reporting Summary

## Data Availability

Summary statistics will be made available through the NHGRI-EBI GWAS Catalog [https://www.ebi.ac.uk/gwas/downloads/summary-statistics] under accession number GCP000102. The direct links to download the summary statistics from GWAS Catalog are as follow: ftp://ftp.ebi.ac.uk/pub/databases/gwas/summary_statistics/GCST90013405, ftp://ftp.ebi.ac.uk/pub/databases/gwas/summary_statistics/GCST90013406, and ftp://ftp.ebi.ac.uk/pub/databases/gwas/summary_statistics/GCST90013407. Genetic association estimates for outcomes considered in Mendelian randomization were obtained from publicly available sources. For coronary heart disease this was the CARDIoGRAMplusC4D Consortium, for ischemic stroke this was the MEGASTROKE Consortium, and for intracerebral hemorrhage this was the International Stroke Genetic Consortium (https://cd.hugeamp.org/downloads.html].
