# Supplementary material for: Genetic analysis in European ancestry individuals identifies 517 loci associated with liver enzymes
Source: Nat Commun. 2021 May 10;12:2579. doi: 10.1038/s41467-021-22338-2 (PMC8110798; doi:10.1038/s41467-021-22338-2)
Supplement: Supplementary file 1 — Supplementary Information [file 41467_2021_22338_MOESM1_ESM.docx]

**Genetic analysis of 753,010 individuals of European ancestry identifies 517 loci for liver enzymes and points to pathways involved in liver and metabolic disorders**

**Supplementary Note 1**

- **The Million Veteran Program (MVP)**

The Million Veteran Program (MVP) is mega-biobank that was launched in 2011 and supported entirely by the Veterans Health Administration Office of Research and Development in the United States (US) of America, in order to develop a genetic repository of US Veterans with additional information from VA electronic health record system and MVP questionnaires to learn how genes, lifestyle, and military exposures affect health and illness. Veterans have been recruited from over 60 Veterans Affairs Medical Centers with informed consent. The specific design and initial demographics of the MVP have been detailed previously ^1^. For clinical and demographic information, electronic health record information from the VA’s Corporate Data Warehouse (CDW) was used. For genetic analyses, DNA extracted from whole blood was genotyped in customized Affymetrix Axiom Array, with further quality control procedures as previously reported ^2,3^ with results from 300,766 Veterans of European ancestry included in this study.

- **Lifelines Study**

Lifelines is a prospective, multi-generational, multi-disciplinary, population-based cohort study of 167,729 residents of the northern part of the Netherland, who were recruited between 2006 and 2009, with a planned follow-up period up to 30 years. Lifelines aims to identify the genetic, environmental, and micro environmental factors, and their interactions associated with aging and the development of complex chronic diseases.  At baseline and during the follow-up, participants are requested to complete several structured, and validated extended self-administered questionnaires consisting of inquiries over demographic, environmental, lifestyle, social, behavioral, psychological, health status, and disease conditions. Physical examinations, aanthropometric measurements, routine biomedical measurements, psychiatric tests, genetic and genomic analyses are being performed for participants during the study. Lifelines is conducted according to the principles of the Declaration of Helsinki and is approved by the medical ethics committee of the University Medical Centre Groningen, The Netherlands. Written informed consent were obtained from all participants.

- **The Rotterdam Study**

The Rotterdam Study (RS) is a prospective population-based cohort study conducted among middle-aged and elderly people in the suburb Ommoord in Rotterdam, the Netherlands. In 1989, 7,983 inhabitants aged 55 and older were recruited in the first cohort (RS-I). In 2000, the RS was extended with a second cohort of 3,011 participants that moved to Ommoord or turned 55 years old (RS-II). In 2006, the third cohort (RS-III) was initiated in which inhabitants aged 45-54 years were invited and included 3,932 participants. Participants have been re-examined every 3-4 years and have been followed up for a variety of diseases. The Rotterdam Study has been approved by the medical ethics committee according to the Population Screening Act: Rotterdam Study, executed by the Ministry of Health, Welfare and Sports of the Netherlands. All participants in the present analysis provided written informed consent to participate and to obtain information from their treating physicians. A more detailed description of the Rotterdam Study can be found elsewhere ^4^. In the Rotterdam Study, blood samples were collected by venipuncture, and immediately frozen (−20°C). Serum GGT, ALT and ALP levels, were determined within two weeks using a Merck Diagnostica kit (Merck, Whitehouse Station, NJ, USA) on an Elan Autoanalyzer (Merck). Non-fasting samples were considered acceptable, as fasting status does not greatly affect serum liver enzyme levels (Ref). All liver biochemistry measurements were obtained in the laboratory of the Department of Epidemiology, Erasmus University Medical Center. For this project, GGT, ALT and AST levels were log-transformed to obtain normal distribution.

**Supplementary Table 1- Liver enzymes data in the Rotterdam Study**

| Rotterdam Study sub sample | N | restriction |
| --- | --- | --- |
| Rotterdam Study 1 (RS1-5) | 3,549 | participants with genotyping and liver enzymes available |
| Rotterdam Study 2 (RS2-3) | 1,014 | participants with genotyping and liver enzymes available |
| Rotterdam Study 3 (RS3-2) | 2,377 | participants with genotyping and liver enzymes available |

- **The Airwave Health Monitoring Study**

The Airwave Health Monitoring Study is a cohort of police force personnel established in 2003 in the UK to study the effect of TETRA, a digital communication system used by police forces. Police forces (who agreed to participate further in the study) underwent clinical assessment and completed comprehensive health assessment questionnaires. By March 2015, over 45,000 participants had attended the health screening ^5^. Age group of the participants ranged between 18 to 65 years of whom two thirds were male and 70% were TETRA users. The Airwave study has collected a broad range of phenotypes including demographics, diet assessments, conventional cardiovascular risk factors, inflammation and metabolic biomarkers, anthropometry, family history of cardiovascular disease, and cardiac/ vascular function. DNA from the blood of the participants has been extracted and the samples have been genotyped using the Illumina Infinium HumanExome-12v1-1 BeadChip Array ^5^ and Affymetrix Biobank array. Quality control procedures and imputation into 1000 genome reference panels have been performed according to the standard protocols of genome-wide association studies ^5^. For ~4,000 participants of the Airwave study, untargeted metabolomics data have been obtained and are available.

- **Northern Finland Birth Cohort 1966**

The Northern Finland Birth Cohort 1966 (NFBC1966) is a prospective follow-up study of children from the two northernmost provinces of Finland born in 1966.^6^ All individuals still living in northern Finland or the Helsinki area (n = 8,463) were contacted and invited for clinical examination. A total of 6007 participants attended the clinical examination at the participants’ age of 31 years. DNA was extracted from blood samples given at the clinical examination (5,753 samples available).^7^ The subset with DNA is representative of the original cohort in terms of major environmental and social factors. Informed consent was obtained from all subjects. For genetic analysis we performed standard sample QC and included 5,402 NFBC1966 participants that were genotyped on an Illumina HumanCNV370DUO Analysis BeadChip. 329,401 variants were included in the imputation scaffold. Variants were imputed to the HRC reference r1.1 2016 on the Michigan Imputation Server.

**Supplementary Note 2**

UK Biobank genotyping was supported by the British Heart Foundation (grant SP/13/2/30111) for Large-scale comprehensive genotyping of UKB for cardiometabolic traits and diseases: UK CardioMetabolic Consortium. The Airwave Health Monitoring Study was funded 2003-2018 by The Home Office (780-tetra) and is currently funded by the Medical Research Council (MR/R023484/1) and the Economic and Social Research Council with additional funding from the National Institute of Health Research Imperial Biomedical Research Centre. P.E. is Director of the Medical Research Council Centre for Environment and Health and acknowledges support from the Medical Research Council (MR/S019669/1, MR/L01341X/1). P.E. is a member of the UK Biobank Steering Committee. P.E. also acknowledges support from the National Institute of Health Research Health Protection Research Units in Chemical and Radiation Hazards, and Environment and Health. P.E. is a UK Dementia Research Institute professor, UK Dementia Research Institute at Imperial College London. The DRI receives its funding from UK Dementia Research Institute Ltd funded by the UK Medical Research Council, Alzheimer’s Society and Alzheimer’s Research UK. P.E. is associate director of Health Data Research UK-London which is funded by HDR UK Ltd from a consortium led by the UK Medical Research Council. This work used the computing resources of the UK MEDical BIOinformatics partnership (UK MED-BIO) which is supported by the Medical Research Council (MR/L01632X/1). R.P. holds a fellowship supported by Rutherford Fund from Medical Research Council (MR/R0265051/1 and MR/R0265051/2). DG is supported by the Wellcome Trust 4i Programme (203928/Z/16/Z) and British Heart Foundation Centre of Research Excellence (RE/18/4/34215) at Imperial College London.

The main replication sample was based on data from the Million Veteran Program, Office of Research and Development, Veterans Health Administration. The content of this manuscript does not represent the views of the Department of Veterans Affairs or the United States Government. This research was also supported by funding from: the Department of Veterans Affairs award I01-BX003362 (P.S.T. and K.M.C), and acknowledged support for this work from the NIH/NIDDK (DK101478, B.F.V.; 1K23DK115897-01, M.S), the NIH/NHGRI (HG010067, B.F.V.) and a Linda Pechenik Montague Investigator award (B.F.V.).

The generation and management of genome-wide association study genotype data for the Rotterdam Study is supported by the Netherlands Organisation of Scientific Research NWO Investments (nr. 175.010.2005.011, 911-03-012). This study is funded by the Research Institute for Diseases in the Elderly (014-93-015; RIDE2), the Netherlands Genomics Initiative (NGI)/Netherlands Organisation for Scientific Research (NWO) project nr. 050-060-810. The work of CMvD is supported by the NGI Center of Medical Systems Biology. We thank Pascal Arp, Mila Jhamai, Marijn Verkerk, Lizbeth Herrera and Marjolein Peters for their help in creating the GWAS database, and Karol Estrada and Maksim V. Struchalin for their support in creation and analysis of imputed data. The Rotterdam Study is funded by Erasmus Medical Center and Erasmus University, Rotterdam, Netherlands Organization for the Health Research and Development (ZonMw), the Research Institute for Diseases in the Elderly (RIDE), the Ministry of Education, Culture and Science, the Ministry for Health, Welfare and Sports, the European Commission (DG XII), and the Municipality of Rotterdam. This Study is further supported by NWO (Vici 918.76.619). The authors are grateful to the study participants, the staff from the Rotterdam Study and the participating general practitioners and pharmacists.

The LifeLines Cohort Study, and generation and management of GWAS genotype data for the LifeLines Cohort Study is supported by the Netherlands Organization of Scientific Research NWO (grant 175.010.2007.006), the Economic Structure Enhancing Fund (FES) of the Dutch government, the Ministry of Economic Affairs, the Ministry of Education, Culture and Science, the Ministry for Health, Welfare and Sports, the Northern Netherlands Collaboration of Provinces (SNN), the Province of Groningen, University Medical Centre Groningen, the University of Groningen, Dutch Kidney Foundation and Dutch Diabetes Research Foundation. The authors wish to acknowledge the services of the Lifelines Cohort Study, the contributing research centres delivering data to Lifelines, and all the study participants.

The Genotype-Tissue Expression (GTEx) Project was supported by the [Common Fund](http://commonfund.nih.gov/GTEx/index) of the Office of the Director of the National Institutes of Health, and by NCI, NHGRI, NHLBI, NIDA, NIMH, and NINDS. The data used for the analyses described in this manuscript (GTEx_Analysis_v7_eQTL) were obtained from the [GTEx Portal](http://www.gtexportal.org/) on 15/02/18.

We gratefully acknowledge all the studies and databases that made GWAS summary data available on LDhub: **ADIPOGen** (Adiponectin genetics consortium), **C4D** (Coronary Artery Disease Genetics Consortium), **CARDIoGRAM** (Coronary ARtery DIsease Genome wide Replication and Meta-analysis), **CKDGen** (Chronic Kidney Disease Genetics consortium), **dbGAP** (database of Genotypes and Phenotypes), **DIAGRAM** (DIAbetes Genetics Replication And Meta-analysis), **ENIGMA** (Enhancing Neuro Imaging Genetics through Meta Analysis), **EAGLE** (EArly Genetics & Lifecourse Epidemiology Eczema Consortium, excluding 23andMe), **EGG** (Early Growth Genetics Consortium), **GABRIEL** (A Multidisciplinary Study to Identify the Genetic and Environmental Causes of Asthma in the European Community), **GCAN** (Genetic Consortium for Anorexia Nervosa), **GEFOS** (GEnetic Factors for OSteoporosis Consortium), **GIANT** (Genetic Investigation of ANthropometric Traits), **GIS** (Genetics of Iron Status consortium), **GLGC** (Global Lipids Genetics Consortium), **GPC** (Genetics of Personality Consortium), **GUGC** (Global Urate and Gout consortium), **HaemGen** (haemotological and platelet traits genetics consortium), **HRgene** (Heart Rate consortium), **IIBDGC** (International Inflammatory Bowel Disease Genetics Consortium), **ILCCO** (International Lung Cancer Consortium), **IMSGC** (International Multiple Sclerosis Genetic Consortium), **MAGIC** (Meta-Analyses of Glucose and Insulin-related traits Consortium), **MESA** (Multi-Ethnic Study of Atherosclerosis), **PGC** (Psychiatric Genomics Consortium), **Project MinE** consortium, **ReproGen** (Reproductive Genetics Consortium), **SSGAC (**Social Science Genetics Association Consortium) and **TAG** (Tobacco and Genetics Consortium), **TRICL** (Transdisciplinary Research in Cancer of the Lung consortium), **UK Biobank**. We gratefully acknowledge the contributions of Alkes Price (the systemic lupus erythematosus GWAS and primary biliary cirrhosis GWAS) and Johannes Kettunen (lipids metabolites GWAS).


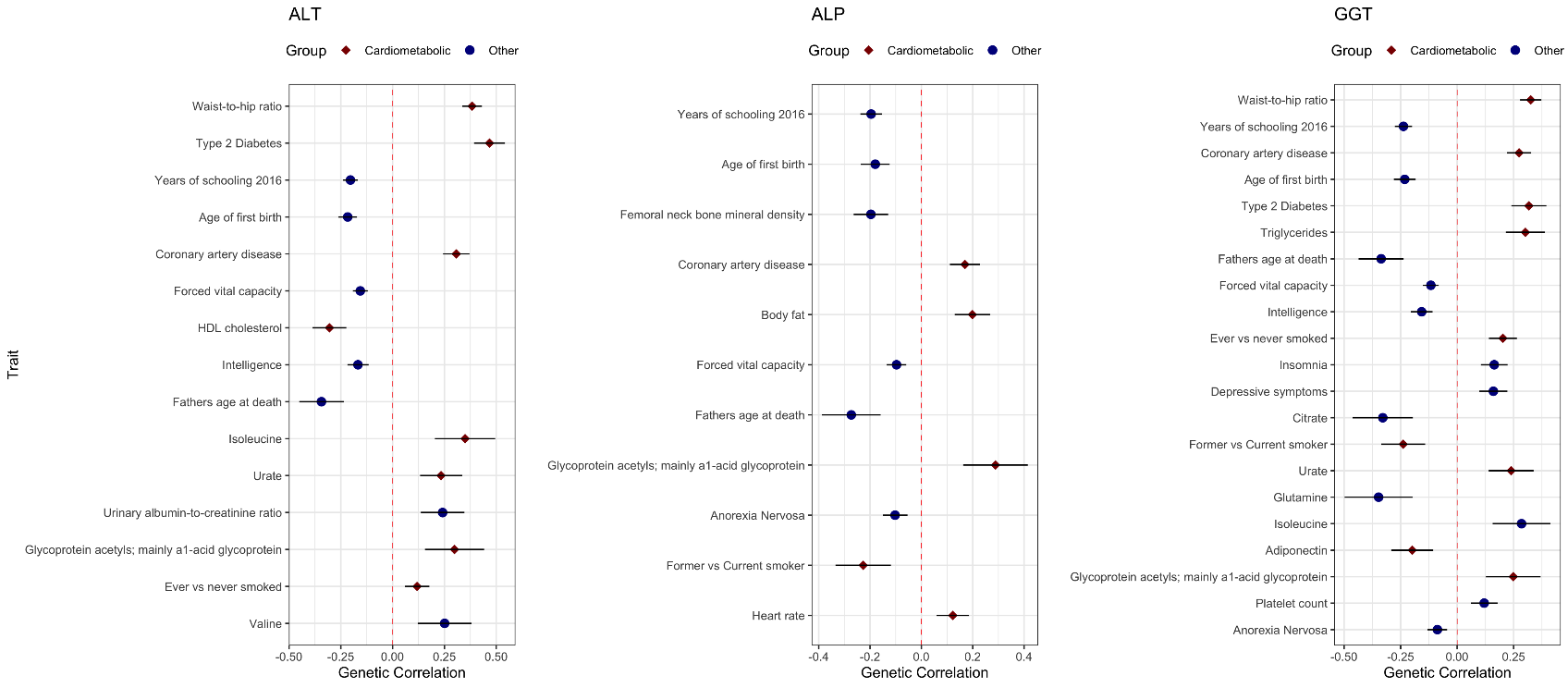


**Supplementary figure 1- Overview of Genetic correlation between ALT, ALP, and GGT SNPs with 257 LD hub traits using discovery stage summary statistics.** Genetic correlation for ALT (left panel), ALP (middle panel), and GGT (right panel) ranked according to *P-*value of the genetic correlation is illustrated.


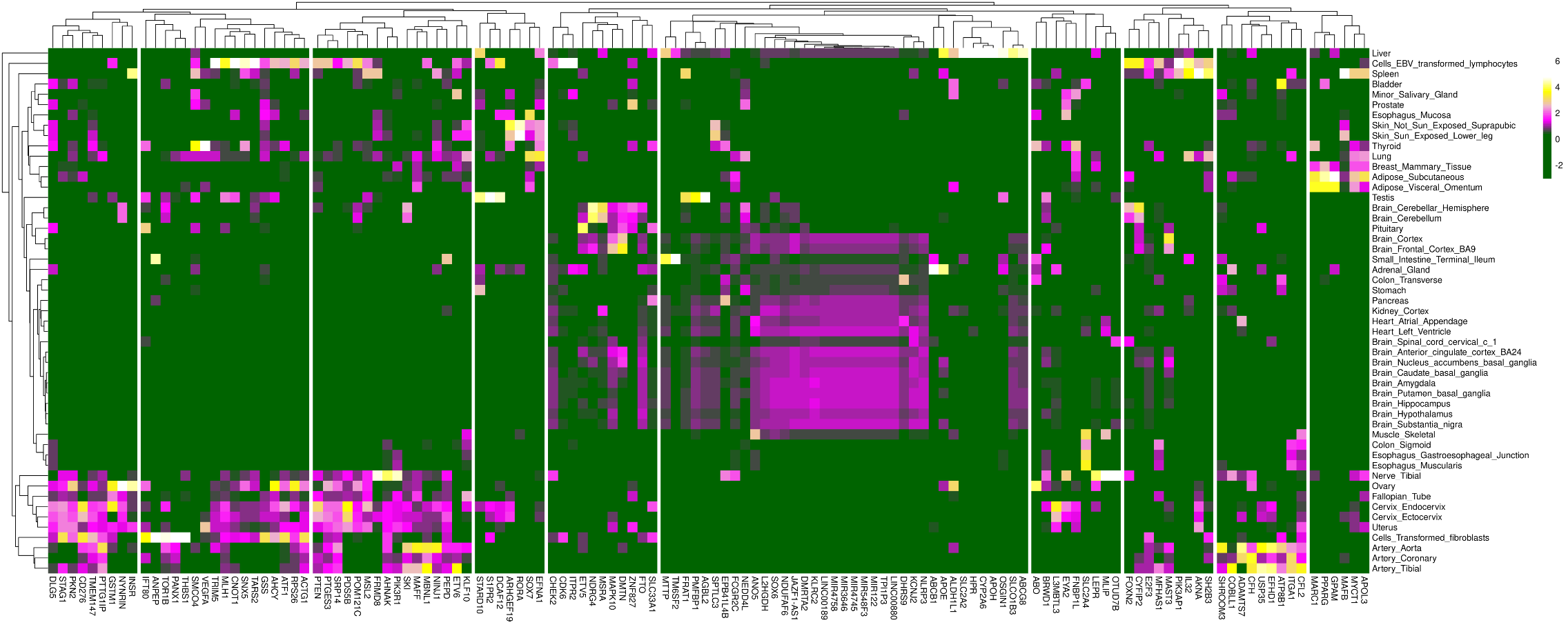


**Supplementary figure 2- Heatmap of median gene expressions for nearest genes to novel ALT SNPs in 51 tissues within GTeX database.** Green represents low expression, Magenta represent medium expression, and yellow and white represent high expression.


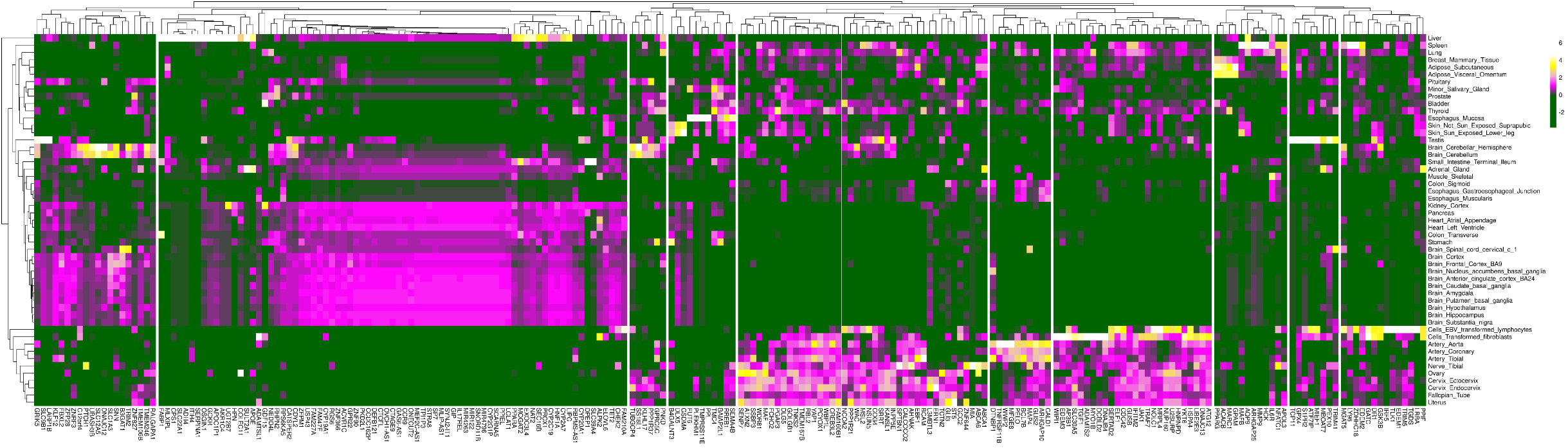


**Supplementary figure 3- Heatmap of median gene expressions for nearest genes to novel ALP SNPs in 51 tissues within GTeX database.** Green represents low expression, Magenta represent medium expression, and yellow and white represent high expression.


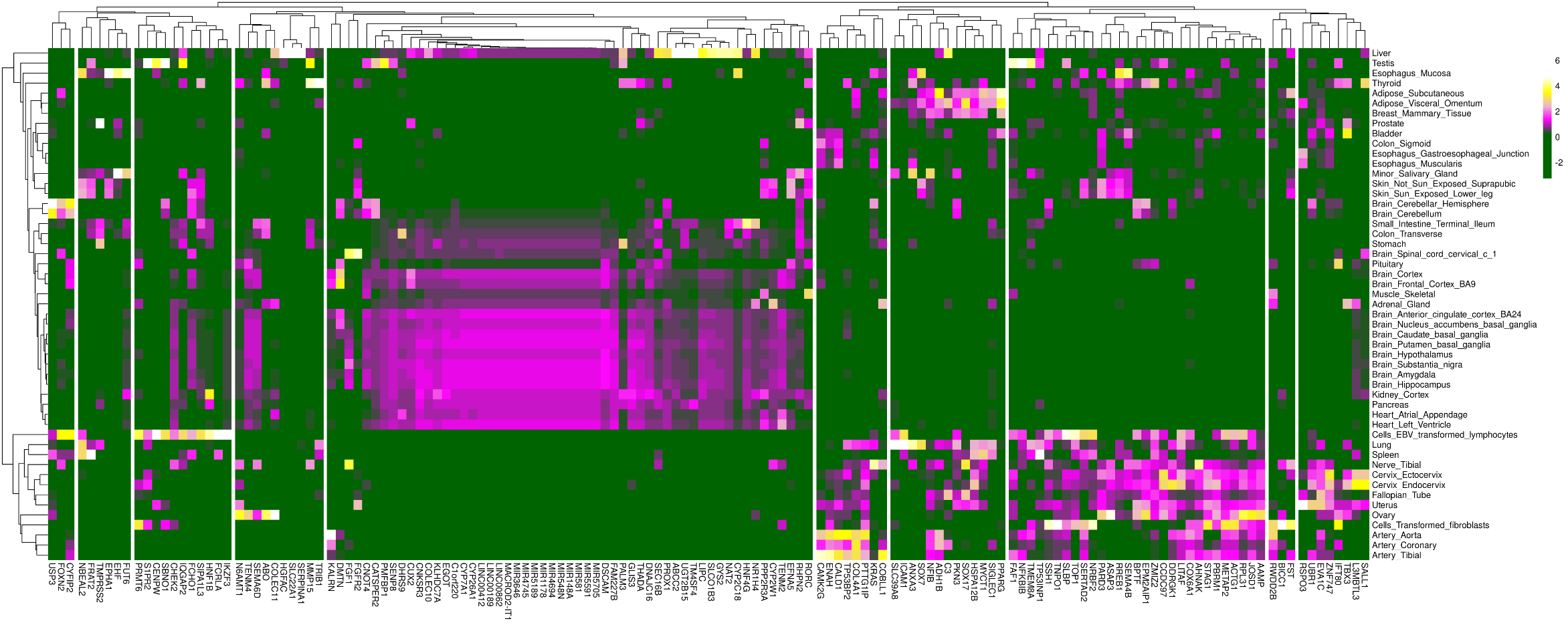


**Supplementary figure 4- Heatmap of median gene expressions for nearest genes to novel GGT SNPs in 51 tissues within GTeX database.** Green represents low expression, Magenta represent medium expression, and yellow and white represent high expression.

**References**

1. Gaziano, J.M. *et al.* Million Veteran Program: A mega-biobank to study genetic influences on health and disease. *J Clin Epidemiol* **70**, 214-23 (2016).

2. Serper, M. *et al.* Validating a non-invasive, ALT-based non-alcoholic fatty liver phenotype in the million veteran program. *PLoS One* **15**, e0237430 (2020).

3. Vujkovic, M. *et al.* Discovery of 318 new risk loci for type 2 diabetes and related vascular outcomes among 1.4 million participants in a multi-ancestry meta-analysis. *Nat Genet* **52**, 680-691 (2020).

4. Ikram, M.A. *et al.* Objectives, design and main findings until 2020 from the Rotterdam Study. *Eur J Epidemiol* **35**, 483-517 (2020).

5. Elliott, P. *et al.* The Airwave Health Monitoring Study of police officers and staff in Great Britain: rationale, design and methods. *Environ Res* **134**, 280-5 (2014).

6. Rantakallio, P. The longitudinal study of the northern Finland birth cohort of 1966. *Paediatr Perinat Epidemiol* **2**, 59-88 (1988).

7. Sovio, U. *et al.* Genetic determinants of height growth assessed longitudinally from infancy to adulthood in the northern Finland birth cohort 1966. *PLoS genetics* **5**, e1000409-e1000409 (2009).

**Supplementary figure 1- Overview of Genetic correlation between ALT, ALP, and GGT SNPs with 257 LD hub traits using discovery stage summary statistics.** Genetic correlation for ALT (left panel), ALP (middle panel), and GGT (right panel) ranked according to *P-*value of the genetic correlation is illustrated.
