# Supplementary material for: Genetic analysis in European ancestry individuals identifies 517 loci associated with liver enzymes
Source: Nat Commun. 2021 May 10;12:2579. doi: 10.1038/s41467-021-22338-2 (PMC8110798; doi:10.1038/s41467-021-22338-2)
Supplement: Supplementary file 2 — Description of Additional Supplementary Files [file 41467_2021_22338_MOESM2_ESM.pdf]

Description of Additional Supplementary Files

File Name: Supplementary Data 1
Description: Characteristics of replication cohorts.

File Name: Supplementary Data 2
Description: Genetic variants associated with ALP within the UKB based on BOLT LMM and GCTA at P<1*10-8.

File Name: Supplementary Data 3
Description: Genetic variants associated with ALT within the UKB based on BOLT LMM and GCTA at P<1*10-8.

File Name: Supplementary Data 4
Description: Genetic variants associated with GGT within the UKB based on BOLT LMM and GCTA at P<1*10-8.

File Name: Supplementary Data 5
Description: Known and novel genetic variants associated with ALP within the meta-analysis of UKB and replication cohorts at P<1×10-9.

File Name: Supplementary Data 6
Description: Known and novel genetic variants associated with ALT within the meta-analysis of UKB and replication cohorts at P<1×10-9.

File Name: Supplementary Data 7
Description: Known and novel genetic variants associated with GGT within the meta-analysis of UKB and replication cohorts at P<1×10-9.

File Name: Supplementary Data 8
Description: Overview of LD score regression between liver enzymes and other traits.

File Name: Supplementary Data 9
Description: Overview of association results for liver enzymes genetic loci and genetically correlated traits in previously published genome-wide association studies.

File Name: Supplementary Data 10
Description: Overview of metabolites associated with liver enzyme SNPs in Airwave.

File Name: Supplementary Data 11.
Description: Overview of liver enzyme SNP cis- eQTL effects in liver

File Name: Supplementary Data 12.
Description: Overview of enriched mammalian phenotypes using DEPICT at False discovery rate < 5% for ALT, ALP, and GGT loci.

File Name: Supplementary Data 13.

Description: Overview of Mendelian randomization analysis for liver enzymes against coronary heart disease and stroke.

File Name: Supplementary Data 14.

Description: Overview of the liver enzyme genetic risk score analysis in the UKB at $P<0.05$.

File Name: Supplementary Data 15.
Description: Overview of the impact of adjustment for metabolic factors on the association between liver enzyme GRS and CVD.
