# Supplementary material for: Genetic analysis in European ancestry individuals identifies 517 loci associated with liver enzymes
Source: Nat Commun. 2021 May 10;12:2579. doi: 10.1038/s41467-021-22338-2 (PMC8110798; doi:10.1038/s41467-021-22338-2)
Supplement: Supplementary file 4 — Reporting Summary [file 41467_2021_22338_MOESM4_ESM.pdf]

nature research

Corresponding author(s): Paul Eliott

Last updated by author(s): 21/07/20

Reporting Summary

Nature Research wishes to improve the reproducibility of the work that we publish. This form provides structure for consistency and transparency in reporting. For further information on Nature Research policies, see Authors & Referees and the Editorial Policy Checklist .

Statistics

For all statistical analyses, confirm that the following items are present in the figure legend, table legend, main text, or Methods section.

n/a	Confirmed	
☐	☒	The exact sample size (*n*) for each experimental group/condition, given as a discrete number and unit of measurement
☐	☒	A statement on whether measurements were taken from distinct samples or whether the same sample was measured repeatedly
☐	☒	The statistical test(s) used AND whether they are one- or two-sided<br>*Only common tests should be described solely by name; describe more complex techniques in the Methods section.*
☐	☒	A description of all covariates tested
☐	☒	A description of any assumptions or corrections, such as tests of normality and adjustment for multiple comparisons
☐	☒	A full description of the statistical parameters including central tendency (e.g. means) or other basic estimates (e.g. regression coefficient) AND variation (e.g. standard deviation) or associated estimates of uncertainty (e.g. confidence intervals)
☐	☒	For null hypothesis testing, the test statistic (e.g. *F*, *t*, *r*) with confidence intervals, effect sizes, degrees of freedom and *P* value noted<br>*Give P values as exact values whenever suitable.*
☒	☐	For Bayesian analysis, information on the choice of priors and Markov chain Monte Carlo settings
☒	☐	For hierarchical and complex designs, identification of the appropriate level for tests and full reporting of outcomes
☐	☒	Estimates of effect sizes (e.g. Cohen's *d*, Pearson's *r*), indicating how they were calculated

Our web collection on statistics for biologists contains articles on many of the points above.

Software and code

Policy information about availability of computer code

Data collection	*Provide a description of all commercial, open source and custom code used to collect the data in this study, specifying the version used OR state that no software was used.*
Data analysis	IMPUTE version 2<br>BOLT-LMM version2.3<br>Genome-wide Complex Traits Analysis (GCTA) version 1.26.0<br>Ingenuity pathway analysis (IPA) version 60467501<br>PLINK2<br>METAL 2016-02-02

For manuscripts utilizing custom algorithms or software that are central to the research but not yet described in published literature, software must be made available to editors/reviewers. We strongly encourage code deposition in a community repository (e.g. GitHub). See the Nature Research guidelines for submitting code & software for further information.

Data

Policy information about availability of data

All manuscripts must include a data availability statement. This statement should provide the following information, where applicable:

- Accession codes, unique identifiers, or web links for publicly available datasets
- A list of figures that have associated raw data
- A description of any restrictions on data availability

Summary statistics of our genome-wide association analysis will be made available via GWAS Catalog website.

Field-specific reporting

Please select the one below that is the best fit for your research. If you are not sure, read the appropriate sections before making your selection.

- [x] Life sciences
- [ ] Behavioural & social sciences
- [ ] Ecological, evolutionary & environmental sciences

For a reference copy of the document with all sections, see nature.com/documents/nr-reporting-summary-flat.pdf

Life sciences study design

All studies must disclose on these points even when the disclosure is negative.

Sample size	437,438 individuals from the UK Biobank study were included in the study. The sample size was determined centrally by the UK Biobank. We used the whole sample of the UK Biobank to ensure 80% statistical power for genetic variants with frequency of >=5% and effect estimates as small as 1.1 IU/L
Data exclusions	Exclusion and inclusion criteria were pre established. We included individuals of European ancestry following quality measures and exclusions (sex discordance, high missingness/heterozygosity). Individuals with non-European ancestry were excluded from the main analysis. We excluded participants who had withdrawn consent, as well as those who were pregnant or unsure of their pregnancy status at baseline. After removing participants of non-European ancestry and individuals with missing values on liver enzymes, 437,438 individuals were left for GWAS of liver enzymes within UK Biobank.
Replication	An additional sample of 315,572 individuals from three large scale cohorts were used for meta-analysis and replication. Overall, 75% of the loci that were put forward for replication were successfully replicated.
Randomization	This was a genome-wide association study using a quantitative trait in a longitudinal study and randomization was not applicable.
Blinding	No intervention was provided in the study and therefore blinding was not applicable.

Reporting for specific materials, systems and methods

We require information from authors about some types of materials, experimental systems and methods used in many studies. Here, indicate whether each material, system or method listed is relevant to your study. If you are not sure if a list item applies to your research, read the appropriate section before selecting a response.

Materials & experimental systems

n/a	Involved in the study	
☒	☐	Antibodies
☒	☐	Eukaryotic cell lines
☒	☐	Palaeontology
☒	☐	Animals and other organisms
☒	☐	Human research participants
☒	☐	Clinical data

Methods

n/a	Involved in the study	
☒	☐	ChIP-seq
☒	☐	Flow cytometry
☒	☐	MRI-based neuroimaging
